# Cell graph neural networks enable the precise prediction of patient survival in gastric cancer

**DOI:** 10.1038/s41698-022-00285-5

**Published:** 2022-06-23

**Authors:** Yanan Wang, Yu Guang Wang, Changyuan Hu, Ming Li, Yanan Fan, Nina Otter, Ikuan Sam, Hongquan Gou, Yiqun Hu, Terry Kwok, John Zalcberg, Alex Boussioutas, Roger J. Daly, Guido Montúfar, Pietro Liò, Dakang Xu, Geoffrey I. Webb, Jiangning Song

**Affiliations:** 1 Biomedicine Discovery Institute and Department of Biochemistry and Molecular Biology, Monash University, Melbourne, VIC 3800 Australia; 2 Institute of Natural Sciences, School of Mathematical Sciences, Key Laboratory of Scientific and Engineering Computing of Ministry of Education (MOE-LSC), and Center for Mathematics of Artificial Intelligence Institute, Shanghai Jiao Tong University, Shanghai, 200240 China; 3 Max Planck Institute for Mathematics in Sciences, Leipzig, 04103 Germany; 4 School of Mathematics and Statistics, The University of New South Wales, Sydney, NSW 2052 Australia; 5 Key Laboratory of Intelligent Education Technology and Application of Zhejiang Province, Zhejiang Normal University, Jinhua, 321004 China; 6 Department of Mathematics, Department of Statistics, University of California, Los Angeles, CA 90095 USA; 7 Faculty of Medical Laboratory Science, Ruijin Hospital, School of Medicine, Shanghai Jiao Tong University, Shanghai, 200025 China; 8 Biomedicine Discovery Institute and Department of Microbiology, Monash University, Melbourne, VIC 3800 Australia; 9 School of Public Health and Preventive Medicine, Monash University, Melbourne, VIC 3004 Australia; 10 Department of Medical Oncology, The Alfred Hospital, Melbourne, VIC 3004 Australia; 11 The Alfred Hospital and Central Clinical School, Monash University, Melbourne, VIC 3004 Australia; 12 Cambridge Centre for AI in Medicine, Department of Computer Science and Technology, University of Cambridge, Cambridge, CB3 0FD United Kingdom; 13 Department of Data Science and Artificial Intelligence, Monash University, Melbourne, VIC 3800 Australia; 14 Monash Data Futures Institute, Monash University, Melbourne, VIC 3800 Australia

**Keywords:** Gastric cancer, Cancer microenvironment, Cancer imaging, Computational biology and bioinformatics

## Abstract

Gastric cancer is one of the deadliest cancers worldwide. An accurate prognosis is essential for effective clinical assessment and treatment. Spatial patterns in the tumor microenvironment (TME) are conceptually indicative of the staging and progression of gastric cancer patients. Using spatial patterns of the TME by integrating and transforming the multiplexed immunohistochemistry (mIHC) images as Cell-Graphs, we propose a graph neural network-based approach, termed *Cell*−*Graph* *Signature* *or* *CG*_*Signature*_, powered by artificial intelligence, for the digital staging of TME and precise prediction of patient survival in gastric cancer. In this study, patient survival prediction is formulated as either a binary (*short-term* and *long-term*) or ternary (*short-term*, *medium-term*, and *long-term*) classification task. Extensive benchmarking experiments demonstrate that the *CG*_*Signature*_ achieves outstanding model performance, with Area Under the Receiver Operating Characteristic curve of 0.960 ± 0.01, and 0.771 ± 0.024 to 0.904 ± 0.012 for the binary- and ternary-classification, respectively. Moreover, Kaplan–Meier survival analysis indicates that the “digital grade” cancer staging produced by *CG*_*Signature*_ provides a remarkable capability in discriminating both binary and ternary classes with statistical significance (*P* value < 0.0001), significantly outperforming the AJCC 8th edition Tumor Node Metastasis staging system. Using Cell-Graphs extracted from mIHC images, *CG*_*Signature*_ improves the assessment of the link between the TME spatial patterns and patient prognosis. Our study suggests the feasibility and benefits of such an artificial intelligence-powered digital staging system in diagnostic pathology and precision oncology.

## Introduction

Gastric cancer (GC) accounted for 768,793 deaths in 2020, representing the fourth deadliest cancer globally^1^. The 5-year survival rate of GC is around 20%^2^. A more accurate prognosis can greatly assist clinical decision-making, especially regarding which patients would benefit from aggressive treatment. The Tumor-Node-Metastasis (TNM) staging system^3^ is the most prevalent cancer staging system primarily used in hospitals and medical centers worldwide, which reflects information on the primary tumor, affected lymph nodes, and metastasis. Many current treatment recommendations and guidelines are based on the TNM stages. However, significant differences in clinical outcomes have been observed in GC patients with the same TNM stage and similar treatment regimens^4–6^. These findings indicate the TNM staging system has limitations and accordingly, cannot be used to accurately predict the prognosis of cancer patients. As such, new strategies that can provide more tailored staging information and improve prognosis predictions are highly desirable.

Recent years have seen numerous data-driven, machine learning-based studies of cancer prognosis. For instance, Yu et al.^7^ introduced prognosis prediction of lung adenocarcinoma and squamous cell carcinoma of stage I, and their model can distinguish the shorter-term survivors from longer-term survivors (*p* = 0.003 and *p* = 0.023). Mobadersany et al.^8^ presented a survival convolutional neural network (SCNN), and their developed histology image-based SCNN reached comparable performance on astrocytomas of grades III and IV with histology grading or molecular subtyping. In another study, Jiang et al.^9^ proposed the GC-SVM classifier as a powerful survival predictor using the data of immunomarkers and could predict the adjuvant chemotherapy benefit of gastric cancer patients with stages II and III. Wulczyn et al.^10^ conducted a survival prediction study involving multiple cancers based on deep learning, and as a result, their model was capable of making significant survival predictions for five out of ten cancers and could effectively stratify cancer patients of stages II and III. Jiang et al.^11^ developed a convolutional neural network-based classifier from H&E images to predict the prognosis of stage III colon cancer patients. Dimitriou et al.^12^ introduced a K-nearest neighbor-based method to predict the mortality of stage II colorectal cancer patients using immunofluorescence images. Although these prognosis prediction studies achieved promising performance using H&E staining histology or immunohistochemistry staining images, they were often restricted to specific subtypes or stages of the corresponding cancers. Moreover, these studies did not consider any spatial information from the tumor microenvironment (TME).

Cell distribution in the TME is not random but is rather associated with the underlying functional state^13–15^. Therefore, the exploration of the TME of cancer samples would offer critical insights into the key spatial patterns associated with the growth, cancer progression, and thus patient prognosis^16^. The recent advent of the multiplexed immunohistochemistry (mIHC) staining technique enables systematic investigation of the TME^17,18^ and supports extraction of enriched spatial information from the TME, including the cell location, cell types, cell and nucleus morphological information, and related optical information^16,19^. Researchers have applied the mIHC technique to analyze the TME of pancreatic cancer and found that spatial distribution of cytotoxic T cells in proximity to cancer cells correlates with increased overall patient survival^16^. Barua et al.^19^ applied a statistical scoring based method, G-cross function, to measure the patterns of two different cell types, such as T-reg and CD8, and found that high infiltration of T-reg in the core tumor area is an independent predictor of worse overall survival (OS) in patients of non-small cell lung cancer. However, these studies only considered the spatial features of limited cell types and only used handcrafted features. Therefore, comprehensive and quantitative methods that assess the relationships between spatial features descriptive of cell distribution and prognosis are currently lacking.

**Fig. 1:**
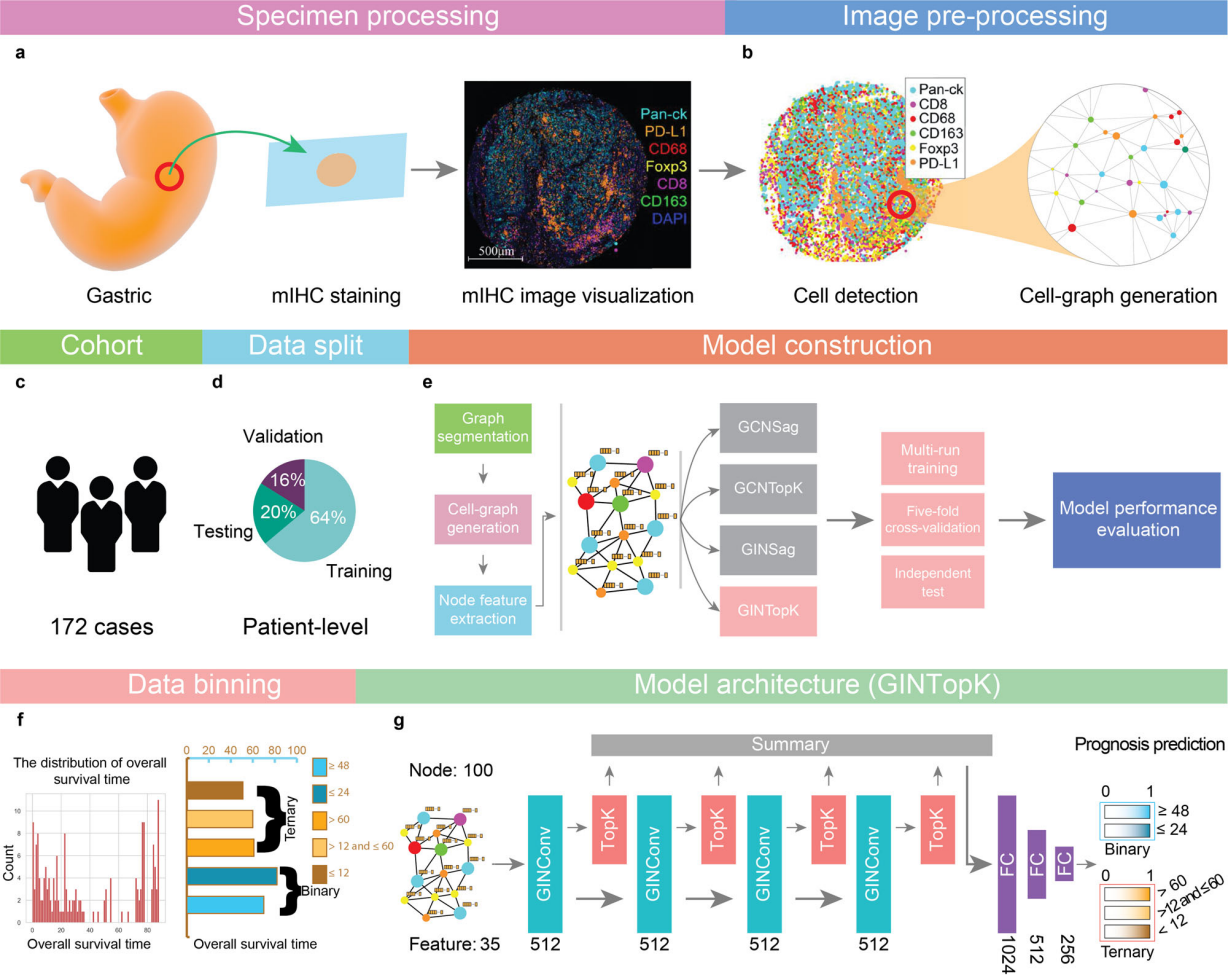
An overall workflow of graph neural network-based prognosis prediction using Cell-Graphs. **a** Specimen processing: the tumor tissues were extracted from gastric cancer, and stained with seven different biomarkers including DAPI, Pan-CK, CD8, CD68, CD163, Foxp3, and PD-L1. **b** Image pre-processing: sub-sampling and cell-graph construction were conducted for image pre-processing. **c** An illustration for the cohort, 172 gastric cancer patients were collected. **d** Data split. The training, validation, and testing datasets were split with the percentages of 64%, 16%, and 20%, respectively. **e** Model construction: four different GNN model architectures, including GCNSag, GCNTopK, GINSag, and GINTopK, were constructed and compared. Multi-run model training, fivefold cross-validation, and independent tests were conducted to evaluate the performance of the constructed GNN models. **f** Data binning: overall survival time ranged from 0 to 88 months, and two data-binning strategies were applied to generate binary- and ternary-class datasets. **g** Model architecture: The four models shared the same architecture but employed different types of convolutional unit and pooling layer, which consists of four consecutive convolutional layer and pooling layer blocks, followed by a summary layer and three fully connected layers, prior to the generation of the final classification outcome. The architecture of the best-performing GINTopK model is illustrated herein, which outperformed the other three model architectures and also achieved the best performance on the test dataset. The corresponding number of hidden layers or feature dimensions is indicated at the bottom of each box. Here, FC stands for “fully connected layer”.

Inspired by the concept of the Cell-Graph^13,20^ and the success of graph neural networks (GNN)^21–23^, especially their applications to the analysis of biology data^24,25^, we hypothesize that intricate spatial distribution information of the TME is informative for the prediction of the OS of GC patients and a GNN model can effectively capitalize on useful patterns generated by Cell-Graphs. To validate this hypothesis, we have developed a novel GNN-based approach for predicting the prognosis of GC patients using Cell-Graph data, which we call the *Cell-Graph Signature* or *CG*_*Signature*_. The overall workflow is illustrated in Figure 1 and Supplementary Fig. 1. In this study, we formulate prognosis prediction as a classification problem by predicting the patient’s survival time interval rather than a continuous time frame or a risk score and develop a workflow to perform the following threefold tasks. Firstly, it extracts comprehensive spatial and morphological information from mIHC images. Secondly, it further uses the extracted spatial information to stratify patients into either binary (*short-term* and *long-term*) or ternary (*short-term*, *medium-term*, and *long-term*) classes. Finally, it conducts the Kaplan–Meier survival analysis to verify the clinical significance of the *CG*_*Signature*_.

*CG*_*Signature*_ represents a powerful survival predictor under comprehensive and extensive benchmarking tests of gastric cancer across all subtypes and stages. Specifically, *CG*_*Signature*_ can effectively stratify short-term, medium-term, and long-term GC survivors at the early diagnosis stage, and achieved area under the receiver-operating characteristic curve (AUROC) values of 0.960 ± 0.01 in terms of binary classification, and 0.771 ± 0.024 to 0.904 ± 0.012 in terms of ternary classification, respectively. In the follow-up survival analysis, *CG*_*Signature*_ outperformed the AJCC 8th edition TNM staging system on the testing cohort in terms of Harrell’s Concordance Index^26^, Hazard Ratio (HR), and *p* value.

## Results

### Clinical characteristics and data-binning of the patient cohort

We collected the gastric cancer patient data between 2008 and 2010 from Shanghai Ruijin Hospital, affiliated with the School of Medicine, Shanghai Jiao Tong University. After removing patients labeled as “lost to follow-up”, we ended up with 172 patients in the cohort for further analysis. All the patients were diagnosed with gastric adenocarcinoma; no neoadjuvant therapies were applied prior to curative gastrectomy. All samples were extracted from surgical samples and were formalin-fixed and paraffin-embedded. The clinical characteristics of this cohort are illustrated in Supplementary Table 1. This cohort contains 124 males, 47 females, and one case without gender information. With respect to the survival status, 113 cases were recorded as “deceased" while 59 patients as “alive". The OS time of the cohort ranges from 0 to 88 months with a median follow-up time of 27 months. Here, the survival time of 0 months indicates the death occurred before the first discharge of the patient from the hospital. The statistical summary of their TNM (the AJCC 8th edition) stages is provided in Supplementary Table 1. In particular, the patient numbers of the TNM stages of I, II, III, and IV are 14, 52, 95, and 3, respectively. Two data-binning strategies were applied to segment the patient OS into binary- or ternary-class datasets. More specifically, patients with OS time shorter than 24 months and longer than 48 months were categorized as short-term, and long-term in the binary-class dataset. The patients whose OS time is between 24 months and 48 months were removed from the training dataset but used in subsequent survival analyses. More details can be found in the section on “Survival analysis and performance comparison with the TNM staging system”. In the ternary-class dataset, patients were classified into short-term, medium-term, and long-term classes, using the thresholds of 12 and 60 months. Here, the thresholds for binary and ternary class data-binning were chosen by considering the relative class balance and the commonly used follow-up periods of 12, 36, and 60 months. To extend the classification margin, we used 24 and 48 months as the thresholds rather than 36 months to divide patients into short-term and long-term classes in binary data binning. We did not optimize the data binning threshold, which can be conducted when more data become available. Model training and subsequent analysis were performed using these two datasets.

### Workflow overview

Figure 1 illustrates an overall workflow and the model architecture of the proposed *CG*_*Signature*_ approach. As shown in Fig. 1a, the mIHC technique was used to stain the GC tissue samples. Specifically, the nuclear counterstain, DAPI, was used for cell nuclei staining, and six antibodies of Pan-CK, CD8, CD68, CD163, Foxp3, and PD-L1 were used as annotation indicators for six different types of cells. After digitalization, cell locations, types, and related optical and morphological features were extracted using the digital pathology software. After this procedure, we obtained the CSV files in which each row corresponds to each cell with the node features shown in Table 1. Based on these CSV files as the input, we developed a workflow (details can be seen in Algorithm 1) to process the raw data and build the GNN-based model to predict the patient OS interval using the features extracted from mIHC images.

**Table 1:** The list of node attributes and their variable types.

Feature name	Feature type
DAPI positive	Boolean
DAPI positive nucleus	Boolean
DAPI positive cytoplasm	Boolean
DAPI nucleus Intensity	Float
DAPI cytoplasm intensity	Float
PD-L1 (Opal 520) positive	Boolean
PD-L1 (Opal 520) positive nucleus	Boolean
PD-L1 (Opal 520) positive cytoplasm	Boolean
PD-L1 (Opal 520) nucleus intensity	Float
PD-L1 (Opal 520) cytoplasm intensity	Float
CD68 (Opal 540) positive	Boolean
CD68 (Opal 540) positive nucleus	Boolean
CD68 (Opal 540) positive cytoplasm	Boolean
CD68 (Opal 540) nucleus intensity	Float
CD68 (Opal 540) cytoplasm intensity	Float
Foxp3 (Opal 570) positive	Boolean
Foxp3 (Opal 570) positive nucleus	Boolean
Foxp3 (Opal 570) positive cytoplasm	Boolean
Foxp3 (Opal 570) nucleus intensity	Float
Foxp3 (Opal 570) cytoplasm intensity	Float
CD8 (Opal 620) positive	Boolean
CD8 (Opal 620) positive nucleus	Boolean
CD8 (Opal 620) positive cytoplasm	Boolean
CD8 (Opal 620) nucleus intensity	Float
CD8 (Opal 620) cytoplasm intensity	Float
Pan-CK (Opal 690) positive	Boolean
Pan-CK (Opal 690) positive nucleus	Boolean
Pan-CK (Opal 690) positive cytoplasm	Boolean
Pan-CK (Opal 690) nucleus intensity	Float
Pan-CK (Opal 690) cytoplasm intensity	Float
Cell area (μm^2^)	Float
Cytoplasm area (μm^2^)	Float
Nucleus area (μm^2^)	Float
Nucleus perimeter (μm)	Float
Nucleus roundness	Float

Each type of feature is comprised of three Boolean variables and two float variables. These Boolean variables were identified by the pathology software based on the float values of Nucleus Intensity and Cytoplasm Intensity of each biomarker. Moreover, five different morphology features were extracted as the node attributes.

The key steps of the workflow are as follows: (1) Image pre-processing: Sub-sampling and Cell-Graph generation were performed at this step. Specifically, each mIHC image was firstly segmented into multiple non-overlapping regions with no more than 100 cells. For each region, we built a graph where each cell was represented as a node and the reciprocal of the Euclidean distance of each cell-cell pair was used to establish edges between them with a distance of fewer than 20 μm. Detailed information can be found in the section “Cell-Graph construction” of Methods. Then, we extracted a total of 35 features (as shown in Table 1) for each cell as the node attributes, including five optical features for each biomarker and five morphological features for each cell. Such generated cell-based graph is referred to as Cell-Graph^13,20^. There are ~90 Cell-Graphs constructed for each mIHC image (for each patient). Cell-Graphs originated from the same mIHC image share the same label with the corresponding patient. (2) Data split: After Cell-Graph construction, the whole dataset was partitioned into the training, validation, and test sets with the ratio of 0.64:0.16:0.20 at the patient level. In addition, we also generated the files for performing fivefold cross-validation by generating five non-overlapping training-validation subsets and evaluating the model performance on these fivefold subsets. (3) Hyperparameter optimization: We utilized the Hyperopt toolkit^27^ from the Ray software package^28^ to tune the hyperparameters of GNN models. The optimized hyperparameters were then used for the follow-up model training and performance evaluation. (4) Model performance evaluation and data visualization: To comprehensively assess the capability and reliability of our GNN model, we evaluate model performance using multi-run model training, fivefold cross-validation, and independent tests. The test results were visualized by generating the receiver-operating characteristic (ROC) curves, confusion matrix, and boxplots of Accuracy, F1-Score, and Matthews Correlation Coefficient (MCC). Performance metrics are defined in Section “Metrics of model performance evaluation” in the Supplementary material.

### Performance benchmarking of different GNN models for prognosis prediction

**Fig. 2:**
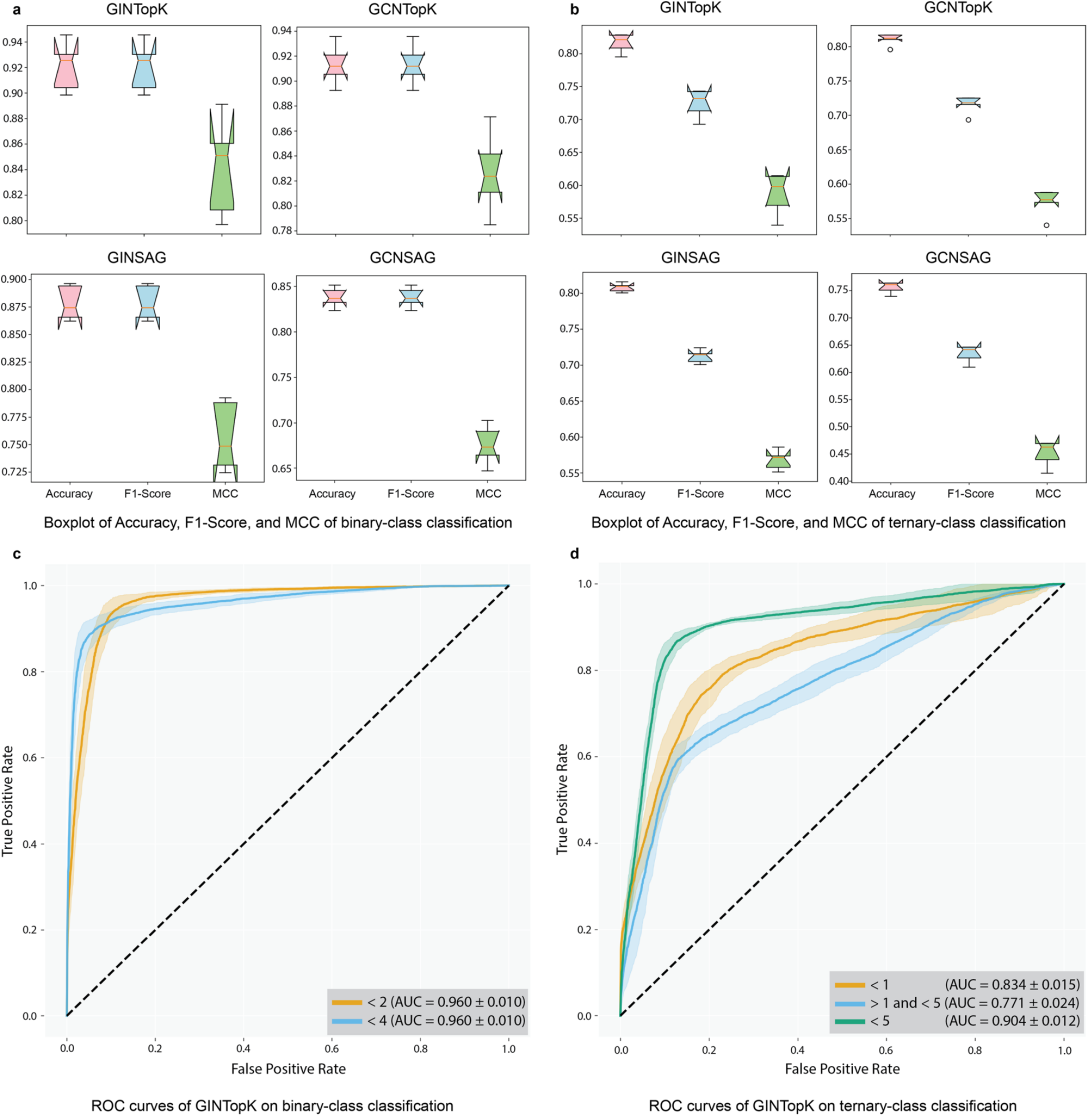
Model performance of four GNNs on fivefold cross-validation. **a**, **b** show the Boxplots of performance metrics of Accuracy, F1-score, and MCC on fivefold cross-validation. **c**, **d** illustrate the ROCs of GINTopK binary- and ternary models on fivefold cross-validation. In the boxplot, the center line marks the mid-point of the data; the top and bottom lines show the maximum and minimum non-outlier data; the upper and lower bounds of the box indicate the third quartile and first quartile of the data; the height of the notch indicates the 95% confidence interval of the median point; small circles represent outliers.

We constructed four different types of GNN models and examined their performance in predicting the OS of gastric cancer patients, including GINTopK, GINSAG, GCNTopK, and GCNSAG. Here, GIN^23^, and GCN^29^ are two graph convolution computational units (differences can be seen in Supplementary Fig. 2), whereas TopKPooling^22,30,31^ and SAGPooling^31,32^ are two graph pooling computational units. The graph convolutional and pooling layers are the core components of the GNN architecture. Fivefold cross-validation was conducted to assess the model of each GNN model on both binary- and ternary-classification tasks. The results are averaged on ten repetitions of fivefold cross-validation for GINTopK on binary classification (as shown in Supplementary Fig. 3) to circumvent the randomness of the model during training. In this procedure, Accuracy, F1-Score, MCC, and AUROC were calculated to evaluate the performance. Figure 2a illustrates the performance results of binary classification on fivefold cross-validation. As we observe, the median values of both Accuracy and F1-score for the four GNNs ranged from 0.83 to 0.92, while the median values of MCC ranged from 0.66 to 0.84, respectively. Figure 2b shows the performance results of ternary classification on fivefold cross-validation. We can see that the ternary-class classification models achieved the median values of Accuracy ranging from 0.76 to 0.82, F1-score from 0.64 to 0.72, and MCC from 0.46 to 0.5, respectively. According to the results shown in Fig. 2a, b, GINTopK slightly outperformed the other three GNN models on both binary- and ternary classifications. Therefore, GINTopK was selected as the best-performing GNN model and employed for subsequent performance benchmarking and survival analysis.

ROC curves of GINTopK on the binary- and ternary-classification tasks are illustrated in Fig. 2c, d, respectively. The binary-class GINTopK model achieved the AUROC value of 0.96 ± 0.01 on fivefold cross-validation. In contrast, the ternary-class GINTopK classifier reached the AUROC values of 0.834 ± 0.015, 0.771 ± 0.024, and 0.904 ± 0.012 for *short-term* (<12 months), *medium-term* (>12 and <60 months), and *long-term* (>60 months) on fivefold cross-validation, respectively (Fig. 2d). Moreover, the performance results of the binary class GINTopK model on ten repetitions of fivefold cross-validation are displayed in Supplementary Fig. 3. We can see that the median values of both Accuracy and F1-score were within the range of 0.90-0.93 (MCC values ranged from 0.80 to 0.86), thereby suggesting the stability of our proposed GINTopK model.

**Fig. 3:**
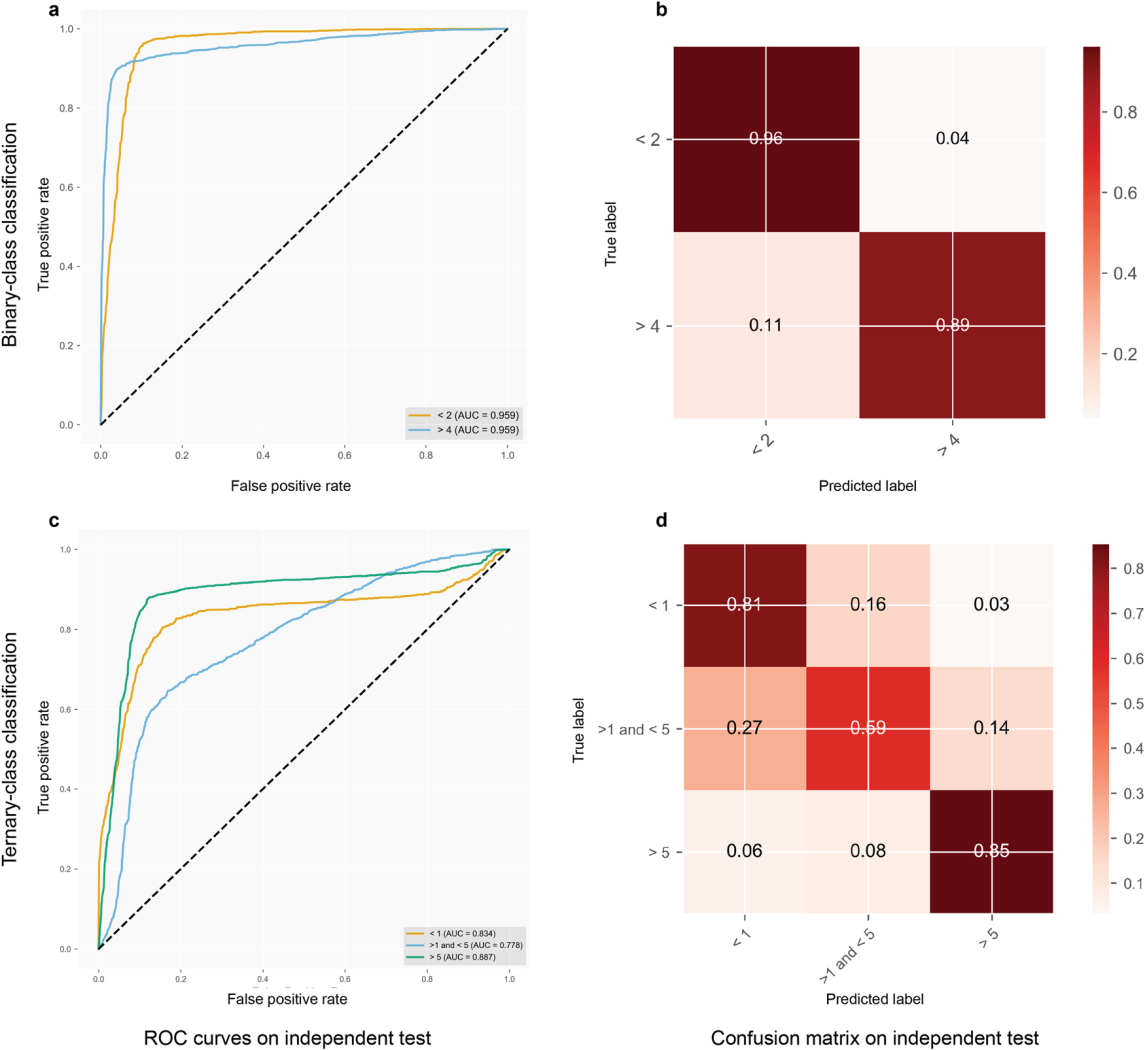
Performance assessment of the GINTopK model in terms of ROC curves and confusion matrix on the independent test. The left column shows the ROC curves of **a** binary- and **c** ternary classification, while the right column displays the confusion matrix of the model predictions on the **b** binary- and **d** ternary classification tasks.

In Fig. 3, the performance results of the GINTopK model on the independent test are visualized using ROC curves and a confusion matrix. It can be seen that the model achieved similar performance to that on fivefold cross-validation in terms of AUROC values on both binary- and ternary-classification tasks. In terms of the confusion matrix, 96% and 89% of the short-term and long-term patients could be accurately predicted using the binary-classification model. The true positive percentages of the ternary-class model were 81%, 59%, and 85%, corresponding to the short-term, medium-term, and long-term classes (Fig. 3).

Taken together, the outstanding performance of the GINTopK model on both cross-validation and independent test indicate that our proposed GNN approach is capable of effectively capturing the underlying prognostic patterns from the well-constructed Cell-Graphs. The captured prognostic patterns by GNN model are characteristic of the spatial information of cell locations and types of the TME, which incorporates more potentially informative features than the TNM staging system.

### Ablation studies and prognostic value of different types of cell features

To examine the effect of node features of different cell types on model performance, we further performed ablation studies to assess the contribution of features to the binary- and ternary-classification performance by removing each type of feature in an iterative manner. Thirty-five node features of seven types were used in this study, including DAPI, Pan-CK, CD8, CD68, Foxp3, PD-L1, and morphological features. We first evaluated the performance of the GNN model trained using all these features, and then, evaluated the performance of the models trained using the remaining features after removing each type of feature from the all-feature set in turn. For each iteration, we trained the models five times with random initialization of the weights using the same dataset and calculated the mean and standard deviation of Accuracy. The results are shown in Table 2, where the feature contribution was measured by the accuracy change compared with that of the all-feature model. Note that when a type of feature is removed, and accuracy increase means that including the feature type reduced accuracy, and an accuracy decrease means that the feature type played an important role in attaining the all-feature accuracy.

**Table 2:** Ablation studies of the major types of features used by the GNN models in both binary and ternary classification.

Feature sets	ACC of binary	ACC of ternary
All-features	0.917 ± 0.012	0.719 ± 0.020
No-DAPI	0.882 ± 0.022	0.710 ± 0.018
No-PD-L1	0.921 ± 0.014	0.718 ± 0.006
No-CD68	0.911 ± 0.011	0.717 ± 0.010
No-FOXP3	0.918 ± 0.017	0.719 ± 0.016
No-CD8	0.910 ± 0.006	0.732 ± 0.023
No-Pan-CK	0.927 ± 0.023	0.714 ± 0.016
No-morphology	0.892 ± 0.009	0.706 ± 0.021

The relative importance and contribution of the features were measured by the accuracy change compared with that of the all-feature model.

According to Table 2, the variant models trained using these feature subsets and all-feature sets achieved comparable Accuracy values in both binary and ternary classifications. In the binary classification, the DAPI features and morphological features made more important contributions to the model performance compared with other types of features (e.g., the Accuracy dropped by 0.035 and 0.025, respectively), which reflects the nucleus differences in optical and morphology of the TME. Thus, the inclusion of these two types of features helped to better distinguish the long-term from short-term patients. In the case of ternary classification, we can see that the GNN models trained without the DAPI and morphology features achieved the lowest Accuracy, which is consistent with the observation in the binary classification. In summary, our proposed GNN model can effectively learn distinguishable spatial features from the TME and further enhance the performance of prognosis stratification by combining the DAPI and morphology features.

### Survival analysis and performance comparison with the TNM staging system

**Fig. 4:**
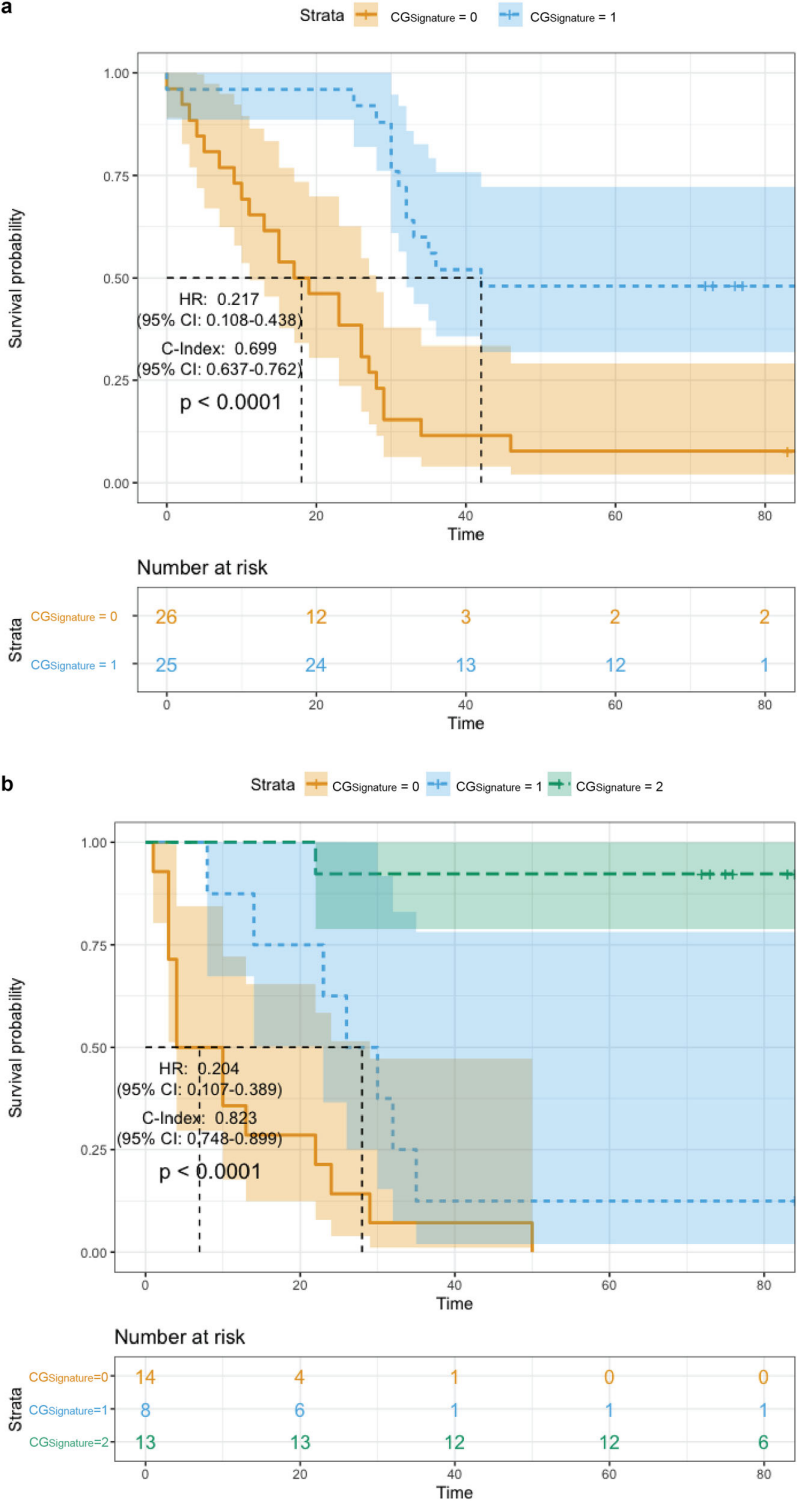
Kaplan–Meier survival analysis of patient overall survival based on the “digital grade” (patient-level predictions) produced by *CG*_*Signature*_. **a** Kaplan–Meier survival analysis results based on the binary-classification. **b** Kaplan–Meier survival analysis results based on the ternary classification.

To further investigate the prognostic values and clinical importance of the predictions produced by *CG*_*Signature*_, we conducted the Kaplan–Meier survival analysis using the patient-level results of both binary- and ternary classifications. For each patient, we first collected the predicted results of all the subsampled Cell-Graphs. Next, we calculated the class percentages of these predictions, and took the class with the maximum percentage as the final patient-level prediction of the corresponding patient. Using these patient-level predicted results (‘digital grade’) of binary classification (with predicted class labels of *CG*_*Signature*_ = 0 and *CG*_*Signature*_ = 1) and ternary classification (with predicted class labels of *CG*_*Signature*_ = 0, *CG*_*Signature*_ = 1, and *CG*_*Signature*_ = 2), we conducted the survival analysis and plotted their Kaplan-Meier curves, shown in Fig. 4. More specifically, when using the binary-class predictions, the median survival time of patient test cohorts predicted as *CG*_*Signature*_ = 0 and *CG*_*Signature*_ = 1 were about 18 months and 42 months, respectively. The HR was 0.217 (95% CI: 0.108–0.438), the C-Index was 0.699 (95% CI: 0.637–0.762), and the *p* value was <0.0001, indicating that *CG*_*Signature*_ has statistically significant prognostic power in separating the two groups of patient cohorts. When using the ternary-class predictions, the median survival time of patient cohorts predicted as *CG*_*Signature*_ = 0 and *CG*_*Signature*_ = 1 were ~7 months and 28 months, respectively. The endpoint survival rate of *CG*_*Signature*_ = 2 was ~92.3% (Fig. 4b). The HR and C-Index were 0.204 (95% CI: 0.107–0.389) and 0.823 (95% CI: 0.748–0.899), respectively, with the *p* value < 0.0001.

We further conducted the univariate and multivariate Cox regression analyses based on the predictions of *CG*_*Signature*_ and the AJCC 8th edition TNM stages. The results are shown in Table 3. In the TNM staging system, there were eight groups of *I*_*A*_, *I*_*B*_, *II*_*A*_, *II*_*B*_, *III*_*A*_, *III*_*B*_, *III*_*C*_, *IV*_*A*_, and *IV*_*B*_. As no patients of stage *IV* were included in the binary-class test cohort and only one patient of stage *IV* was included in the ternary-class test cohort, we excluded the patients of stage *IV* and those without OS information. Finally, 51 patients (including 20 uncategorized patients) and 35 patients were retained for binary- and ternary-class survival analysis, respectively. The detailed statistical information of the testing cohorts can be found in Supplementary Table 2.

**Table 3:** Univariate and multivariable Cox regression analysis of overall survival (Cox proportional hazards regression model) based on the predictions of binary- and ternary classification by *CG*_*Signature*_.

		Univariate analysis			Multivariate analysis		
	Variable	C-Index^1^ (95% CI)	HR^2^ (95% CI)	*p* value	C-Index^1^ (95% CI)	HR^2^ (95% CI)	*p* value
Binary-class test cohort	pT	-	-	-			0.363
	pN	-	-	-			0.378
	TNM-2^3^	0.659 (0.577–0.740)	5.276 (2.147–12.966)	<1e-4^***^			0.998
	TNM-3^4^	-	-	-			0.998
	TNM-6^5^	0.714 (0.623–0.805)	1.873 (1.388–2.529)	8.1e-4^***^			0.135
	${C{G}_{Signature}}^{5}$	0.699 (0.637–0.762)	0.217 (0.108–0.438)	<1e-4^***^	0.804 (0.728–0.881)	0.037 (0.003–0.422)	0.007^**^
	*CG*_*Signature*_ + TNM-2	0.740 (0.661–0.819)	2.412 (1.650–3.525)	<1e-4^***^			0.146
Ternary-class test cohort	pT	-	-	-			0.097
	pN	-	-	-			0.053
	TNM-2^3^	-	-	-			0.998
	TNM-3^4^	0.632 (0.510–0.753)	3.169 (1.335–7.522)	0.019^*^			0.998
	TNM-6^6^	0.681 (0.535–0.827)	1.708 (1.212–2.407)	0.028^*^			0.066
	${C{G}_{Signature}}^{7}$	0.823 (0.748–0.899)	0.204 (0.107–0.389)	<1e-4^***^	0.883 (0.820–0.947)	0.190 (0.087–0.414)	2.94e-05^***^

^1^C-Index: concordance index; ^2^HR: Hazard ratio; ^3^I+II vs. III; ^4^I vs. II vs. III; ^5^I, IIA, IIB, IIIA, IIIB, IIIC; ^6^Low vs. high; ^7^Low vs. medium vs. High.

The classification results were compared with Harrell’s Concordance Index (C-Index), Hazard Ratio (HR), and *p* value. For the convenience of survival analysis comparison, the variables of TNM stages were regrouped into TNM-2 (I+II vs. III), TNM-3 (I vs. II vs. III), and TNM-6 (I, IIA, IIB, IIIA, IIIB, IIIC), while “*CG*_*Signature*_+TNM-2" denotes a four-class variable by combining the classes of TNM-2 and binary-class *CG*_*Signature*_.

To make a fair comparison, three specific criteria were adopted to aggregate the TNM stages into TNM-2 (*I*, *II* vs. *III*), TNM-3 (*I* vs. *II* vs. *III*), and TNM-6 (*I* vs. *II*_*A*_ vs. *II*_*B*_ vs. *III*_*A*_ vs. *III*_*B*_ vs. *III*_*C*_). The survival analysis results are provided in Table 3 and Figs. S4–S9. According to the univariate analysis results shown in Table 3 and Supplementary Fig. 4, the C-Index of the binary-class *CG*_*Signature*_ was 0.699 (*p* value < 0.0001), outperforming TNM-2 with an increase of 0.04. We further combined the TNM-2 with binary-class *CG*_*Signature*_ for survival analysis (Supplementary Fig. 6), which achieved the highest C-Index of 0.748 (*p* value < 0.0001), which was higher than TNM-6 by 0.034 (Supplementary Fig. 5). In ternary-class univariate Cox regression analysis, we compared the results of TNM-3, TNM-6, and the ternary-class *CG*_*Signature*_. More specifically, C-Index of the ternary *CG*_*Signature*_ was 0.823 (*p* value < 0.0001, Fig. 4b), which was superior to the TNM-3 (Supplementary Fig. 8) and TNM-6 (Supplementary Fig. 9) with an increase of 0.191 and 0.142, respectively. Results of multivariate Cox regression analyses on both binary- and ternary-class test cohorts indicate that our proposed *CG*_*Signature*_ (i.e., the only one with *p* value < 0.05) can serve as an independent risk factor compared with other factors derived from TNM stages (all had *p* value > 0.05). Univariate and multivariate analyses demonstrate the *CG*_*Signature*_ is capable of discriminating and stratifying gastric cancer patients into groups of different prognoses better than the TNM staging system. Moreover, we note that the prognostic power can be even further enhanced by integrating the *CG*_*Signature*_ predictions and the TNM stages for survival analysis, such as the *CG*_*Signature*_ + *TNM* − 2 in Table 3 and Supplementary Fig. 6.

To summarize, by combining the spatial information from the mIHC images, *CG*_*Signature*_ has demonstrated outstanding performance in survival analysis and achieved a better or at least comparable performance when compared with the TNM staging system. The results suggest that effective prognostic features can indeed be captured by *CG*_*Signature*_, which suggests a powerful method complementary to the current TNM staging system.

### Framelet decomposition for cell-graph

To examine the capacity of Cell-Graph to capture useful spatial features from mIHC images, we conducted a framelet decomposition on the whole mIHC images. The framelet transforms (including framelet decomposition and reconstruction) have proved an important tool for distilling multi-resolution information in low-pass and high-passes from the graph data^33–37^.

We extracted low-pass and high-pass information of six types of features, corresponding to six different biomarkers DAPI, PAN-CK, CD8, CD68, FOXP3, and PD-L1. Tables S3–S11 show the low-pass and high-pass coefficients of the framelet decomposition on mIHC images of short-term, medium-term, and long-term survivors on the entire mIHC images. For the selected samples, no significant differences were observed from the low-pass channel. However, major differences can be observed from the high-pass channel on the selected samples. More specifically, remarkable signal differences can be seen from the high-pass channel-1 and channel-2 in terms of the features of Cell Area and Nucleus Perimeter (summarized in Supplementary Tables 6–11). These differences highlight the important prognostic value of cell morphological information of the TME, which is consistent with the prognostic value of different types of cell features.

## Discussion

In this study, we developed a GNN-based approach, Cell-Graph Signature (*CG*_*Signature*_), which is capable of predicting the prognosis of gastric cancer patients from Cell-Graphs extracted from mIHC images. Extensive benchmarking tests on multi-run model training, fivefold cross-validation, and independent tests demonstrate that *CG*_*Signature*_ can accurately predict the prognosis on both binary- and ternary-class classification tasks. We designed and compared the performance of four different GNN architectures, including GINSag, GCNTopK, GCNSag, and GINTopK. As a result, GINTopK achieved the best performance when compared with the other three GNN architectures (GINSag, GCNTopK, and GCNSag) on the same data sets. Feature ablation studies showed that the nucleus optical feature (DAPI) and cell morphological features are essential node features and contributed most to the prognosis prediction, which indicates the potential pivotal roles of nuclear and cell morphology in gastric cancer progression. In survival analysis, *CG*_*Signature*_ clearly outperformed the AJCC 8th TNM staging system in terms of C-Index (0.823, 95% CI: 0.748–0.899) using the ternary-classification model. In particular, we notice that *CG*_*Signature*_ achieved better or comparable performance with the TNM staging system when using the binary-classification model. These results of survival analysis indicate that *CG*_*Signature*_ provides more prognostic power than the existing TNM staging system and can help pinpoint patients who may benefit from more tailored and personalized therapy. Moreover, wavelet decomposition results suggest that Cell-Graphs can indeed capture certain important spatial features informative for classifying patient survival. Although many previous studies of prognosis prediction also achieved promising results, the majority of such studies were only limited to a specific subtype or stage of cancer. Nevertheless, in this study, we show that the proposed *CG*_*Signature*_ method is applicable to gastric cancer patients of all subtypes across all TNM stages. Moreover, *CG*_*Signature*_ achieved better performance when stratifying test patient cohorts into different groups of prognosis, which has proven a powerful prognostic predictor for gastric cancer.

One caveat of the current study is that we could only obtain mIHC image data for a limited number of patients, and accordingly, the performance of the *CG*_*Signature*_ was only benchmarked on a gastric cancer patient dataset with a limited size. In addition, we were not able to collect a sufficient number of stage IV patients, and thus the model performance needs further verification and improvement when more data become available in the future. Thus, in future studies, it would be important to evaluate the performance of GNNs based on Cell-Graph data from mIHC images in much larger and/or multi-center patient cohorts, as well as additional tumor types (in addition to gastric cancer), when more data become available. Exploration of the prognostic value of the *CG*_*Signature*_ method on datasets of other cancer types would surely be needed to verify its utility and capability. Additionally, future extension of the capability of *CG*_*Signature*_ by using whole-slide images and other biomarkers in mIHC/mIF staining, for example, holds great potential for a more comprehensive analysis of the TME^17^; this will in turn serve to better inform the training of more accurate GNN models. The continuing development of cutting-edge, robust, and broadly applicable Cell-Graph-based biomarker discovery algorithms is valuable and desirable to better inform and transform the medical care of cancer patients.

## Methods

### Dataset

The gastric cancer samples were collected and stained with mIHC technique and prepared as two batches of tissue microarray^38^, in which all the samples were arranged in the matrix configuration. Then the two tissue microarrays were scanned by a digital microscope (brand: Vectra Polaris) under the magnification of ×40 with each pixel representing 0.5 μm. Totally, 181 mIHC images of cancer tissues were curated as the initial datasets. After excluding patients whose follow-up data were not available, 172 mIHC images were retained and used for model training and benchmarking. The OS time of the patients ranges from 0 to 88 months, as shown in Fig. 1f. Detailed clinical characteristics and a statistical summary of the cohort are provided in Supplementary Table 1. Fifty-nine patients were still alive at the time of the last follow-up. All the images were stained using multiplexed immunohistochemistry of seven colors and reagents to identify the specific cell types. In this study, cells were stained with antibodies of Pan-CK, Foxp3, CD8, PD-L1, CD68, CD163, and DAPI. Detailed information on these antibodies can be found in Supplementary Table 12. The dataset was randomly partitioned into the training, validation, and test subsets with the ratios of 0.64, 0.16, and 0.20 at the patient level. In addition, datasets for fivefold cross-validation were also prepared. The gastric cancer patient cohort was obtained from Shanghai Jiao Tong University, Ruijin Hospital. This study was approved by the Ethics Committee of Ruijin Hospital, Shanghai Jiao Tong University School of Medicine (ID: 2021-194). Written informed consent was obtained from all patients.

### Label generation

In this study, the survival prediction was formulated as a classification problem in the form of either binary- or ternary classification. To explore the prognostic value of the Cell-Graphs extracted from the gastric cancer TME, the survival time of the cohort was categorized into two and three classes, and used as labels for training binary- and ternary-classification models based on GNNs. In binary classification, 82 patients with a survival time of fewer than 24 months were annotated as short-term while 70 patients with a survival time of longer than 48 months were annotated as long-term. 20 patients with survival times between 24 and 48 months were removed from the training data set, and denoted as uncategorized patients. For the ternary classification, 12 months and 50 months were, respectively, used as the thresholds to divide patients into short-, medium-, and long-term, with the corresponding patient numbers of 51, 60, and 61, respectively.

### Cell segmentation

After digitization, the mIHC images were pre-processed using the pathology software HALO (Indica Labs) for cell segmentation and feature extraction. The extracted information was subsequently saved as a CSV file in which each row represents the features of a cell (as shown in Table 1), including the cell locations, optical features of stained cells, and morphology features. Thirty-five such features were selected as the node features for each cell. Detailed information can be found in the “Node attributes” section.

### Sub-sampling

Each mIHC staining image contains around 7000~13,000 cells. In particular, we conducted the sub-sampling when generating the Cell-Graphs. By treating each cell as a node in the Cell-Graph, we limited the graph size to no more than 100 nodes. A non-overlap sliding window was then applied to extract the local regions that contained ~100 cells from the mIHC images. As a result, we obtained 16951 Cell-Graphs, which would be used for GNN model training and testing. The extracted Cell-Graphs from one mIHC image was annotated with the same label as that of the corresponding mIHC image. The performance of the GNN models was firstly assessed at the Cell-Graph level; After that, the prediction outputs of all Cell-Graphs were aggregated to generate the votes for the final prediction outcome at the patient level.

### Cell-Graph construction

According to the previous study on the TME^19^, we assumed that the maximum effective distance was 20 μm between immune and tumor cells [], which is equivalent to 40 pixels in the magnification of this study. We calculated the Euclidean distance between any pair of cells, and used this distance to define the edge weight between them according to the equations (1) and (2) shown below.

For the *i*th and *j*th cells with Cartesian coordinates (*x*_*i*_, *y*_*i*_) and (*x*_*j*_, *y*_*j*_) (which use pixel as the unit) in the same mIHC image, their Euclidean distance can be calculated as follows:

1 $$d(i,j)=\sqrt{{({x}_{i}-{x}_{j})}^{2}+{({y}_{i}-{y}_{j})}^{2}}.$$

The weight between the *i*th and *j*th cells is assigned as follows:

2 $${w}_{i,j}:= \left\{\begin{array}{ll}40/d(i,j),d(i,j)\le 40\,{{\mbox{pixel}}}\,,\\ 0,\quad\qquad\;\,{{\mbox{otherwise}}}\,.\end{array}\right.$$

where 0 denotes that there is no interaction between the cell *i* and *j*. After sub-sampling, a number of Cell-Graphs (up to 100 nodes) were extracted and annotated, with the weight (2) of the edge between a given pair of cells.

### Node attributes

GNN is a powerful deep learning approach that can efficiently extract features from graph-structured data. In the present study, we focused on distilling five morphology features and 30 optical features generated by six staining biomarkers as the attributes of the node for each cell, including DAPI, PAN-CK, CD8, CD68, FOXP3, and PD-L1. The five morphology features include cell area, cytoplasm area, nucleus area, nucleus perimeter, and nucleus roundness. The optical features of each biomarker are comprised of the positive, positive nucleus, positive cytoplasm, nucleus intensity, and cytoplasm intensity. As a result, a total of 35 features were extracted for each cell. The detailed list of the features and their data types is listed in Table 1. All the features were linearly normalized to the range of [0, 1] prior to training the GNN models.

### Architecture of the designed GNNs

Graph-structured data are usually represented in the form of (*x*_*i*_, *A*_*i*_), where *x*_*i*_ denotes the feature of the node for the *i*th graph sample while *A*_*i*_ represents its adjacency matrix. A GNN has a similar network architecture to that of the traditional convolutional neural network. To address the classification task in this study, we designed the GNN model architecture of *CG*_*Signature*_, which includes four computational units, each with two-layer *graph convolution* plus one-layer *graph pooling* followed by three-layer fully connected layers (MLP), before generating the prediction output (Fig. 1).

The graph convolutional layer is responsible for extracting an array of features from the last output array, which mimics the role of CNN convolution. It changes the dimension *d* of the feature array but does not change the number of nodes *N*_*i*_. The output of graph convolutional layers is passed on to the graph pooling which compresses the node number by a fractional proportion while in this process usually the key structural information and node features are preserved. The MLP readout will then output the label class. Graph convolution communicates the structural information of the data to the deep network model via the message passing between the neighborhood nodes, which contributes as the key to successfully capturing the geometric feature of the data. In this work, we adopted the GINConv^23^ as the graph convolution and TopKPool^22^ as the graph pooling method, respectively. The convolutional layer for GIN can be aggregated by

3 $${{{{\bf{X}}}}}^{{{{\rm{output}}}}}={{{\rm{MLP}}}}\left(\left({{{\bf{A}}}}+(1+\epsilon )\cdot {{{\bf{I}}}}\right)\cdot {{{{\bf{X}}}}}^{{{{\rm{in}}}}}\right),$$

where ${{{{\bf{X}}}}}^{{{{\rm{in}}}}}\in {{\mathbb{R}}}^{N\times d}$ is the *d*-feature matrix on the nodes of the graph with *N* nodes for the input layer, and $A\in {{\mathbb{R}}}^{N\times N}$ is the adjacency matrix of the graph. *W* is the filter weight parameter matrix with the size of *m* × *n* to be learned by the GNNs, where *n* is the number of hidden neurons. GINConv is a special neural message passing operator for GNN aggregation.

Our GNN model was trained by connecting multiple layers of graph convolution activated by a ReLU (Rectifier Linear Unit)^39^. The graph pooling, which is used between two consecutive layers, serves to reduce the dimensionality of the feature map so that the network has appropriate amounts of parameters to circumvent over-fitting^40^. Here we used *TopKPooling*^22^ for graph pooling.

There exist different types of GNN models in the machine learning literature^41^. Specifically, we tested the performance of the GINConv+TopKPool model with the other three popular GNN models, i.e., GINConv+SAGPool, GCNConv+TopKPool, and GCNConv+SAGPool. The results showed that the chosen model (GINConv+TopKPool) achieved the highest AUROC value and stable training performance. Refer to Figure 2a, b for a detailed illustration of the results.

### Hyperparameter optimization

We fine-tuned the hyperparameters for the GNN models with the assistance of HyperOPT^27^ and Ray^28^, where the network architecture and batch size were fixed. The hyperparameters were searched within the range as shown in Table 4. More specifically, the best-performing model used the following hyperparameters: learning rate 5 × 10^−4^, weight decay rate 10^−4^, number of hidden neurons 512, pooling ratio 0.5, number of hidden layers 4, batch size 256, and maximal number of epochs 200 with the early stopping strategy.

**Table 4:** Search space for hyperparameters of GNN models.

Hyperparameter	Searching space
Learning rate	10^−4^, 5 × 10^−4^, 10^−3^
Weight decay (*L*_2_)	10^−4^, 5 × 10^−4^, 10^−3^
Hidden units	256, 512
Pooling ratio	0.5, 0.65, 0.75

### Prediction aggregation to assess the patient-level performance

The model performance was evaluated at the Cell-Graph level. After the model was optimized, the patient-level performance of the model was calculated by aggregating the prediction results produced by the optimized model. In particular, we fed Cell-Graphs of the test dataset to the optimal model to predict the label for each of them. Since hundreds of Cell-Graphs were subsampled from the mIHC images of the patient, hundreds of the predictions were also made for a given patient. To generate the patient-level prediction for a patient, we calculated the proportion of Cell-Graphs belonging to a specific class, and then classified the patient as the group that received the largest proportion of the Cell-Graphs.

### Framelet analysis to facilitate interpretation of the model prediction

From the mathematical perspective, the *framelet system*^33–35,37^ refers to a set of functions that provide a multi-scale representation of graph-structured data, which has a similar property to the traditional wavelets in the Euclidean space. Using the framelet transforms, we can decompose the graph features into low-pass and high-pass frequencies as the extracted features to train network models, via the framelet-based graph convolution.

Suppose ${\{({\lambda }_{\ell },{u}_{\ell })\}}_{j = 1}^{N}$ are the pairs of the eigenvalue and eigenvector for the graph Laplacian ${{{\mathscr{L}}}}$ of a graph ${{{\mathscr{G}}}}$ with *N* nodes. The (undecimated) framelets at the *scale level* *j* = 1, …, *J* for graph ${{{\mathscr{G}}}}$ with the above scaling functions can be defined, for *n* = 1, …, *r*, as follows:

4 $$\begin{array}{ll}{\varphi }_{j,p}(v)=\mathop{\sum }\limits_{\ell =1}^{N}\hat{\alpha }\left(\frac{{\lambda }_{\ell }}{{2}^{j}}\right)\overline{{u}_{\ell }(p)}{u}_{\ell }(v)\\ {\psi }_{j,p}^{n}(v)=\mathop{\sum }\limits_{\ell =1}^{N}\widehat{{\beta }^{(n)}}\left(\frac{{\lambda }_{\ell }}{{2}^{j}}\right)\overline{{u}_{\ell }(p)}{u}_{\ell }(v),\end{array}$$

where *φ*_*j*,*p*_ and ${\psi }_{j,p}^{r}$ are the low-pass and high-pass framelets translated at the graph node *p*. In the framelet analysis above, we have shown the low-pass and high-pass *framelet coefficients* *v*_*j*,*p*_ and ${w}_{j,p}^{r}$ for a signal *f* on graph ${{{\mathscr{G}}}}$. They are the projections 〈*φ*_*j*,*p*_, *f*〉 and $\langle {\psi }_{j,p}^{r},f\rangle$ of the graph signal onto framelets at the scale *j* and node *p*. The construction of the framelet system and the framelet transforms rely on the filter bank (a collection of filters) to calculate framelet coefficients. Here we used the filter bank of the Haar-type filters for the experiments^33,37^. The dilation factor is 2^*j*^ with the *dilation* (base) 2 for a natural number *j*, where *j* indicates the scale level and 2^*j*^ is the scale of the framelet. A bigger value of *j* indicates that the corresponding framelet coefficient carries more detailed information about the graph signal.

The above framelet system is a *tight frame*, which provides an exact representation of any *L*_2_ function on the graph. This guarantees that the framelet coefficients have a unique representation of a graph signal. Accordingly, the framelet coefficients can fully reflect the feature of the signal. Moreover, the coefficients decompose the signal at multi scales and can be used to observe whether a particular scale or the high-pass or low-pass frequencies contain a more important feature of the data.

### Reporting summary

Further information on research design is available in the Nature Research Reporting Summary linked to this article.

## Supplementary information

Supplementary file

REPORTING SUMMARY

## Data Availability

The data used for the main analyses presented here is available for non-commercial use and can be accessible by request.

## Code availability

All the related scripts and code are publicly available and can be downloaded at https://github.com/docurdt/Cell-Graph_Signature.git.

## Materials availability

Hardware: all the experiments were performed using PyTorch Geometric^42^ on a server with Intel(R) Core(TM) i9-9820X 230 CPU 3.30GHz, NVIDIA GeForce RTX 2080Ti, and NVIDIA TITAN V GV100.
