# Supplementary material for: Cell graph neural networks enable the precise prediction of patient survival in gastric cancer
Source: NPJ Precis Oncol. 2022 Jun 23;6:45. doi: 10.1038/s41698-022-00285-5 (PMC9226174; doi:10.1038/s41698-022-00285-5)
Supplement: Supplementary file 1 — Supplementary file [file 41698_2022_285_MOESM1_ESM.pdf]

Supplementary Methods

Pseudo algorithm and workflow

In this section, we describe the sub-sampling procedure of Cell-Graphs from a mIHC image based on the following Algorithm 1 and Figure 1. The node number of the Cell-Graph was set to 100, corresponding to 100 cells that were included in a Cell-Graph. In the magnification of the image scanning, each pixel represents 0.5 μm.

Algorithm 1: Algorithm of Cell-Graph generation

Read a CSV file of one mIHC image;
Set node number: $node\_num \leftarrow 100$;
Obtain the cell number: $cell\_num \leftarrow len(dataframe)$;
Calculate the number of Cell-Graphs: $graph\_num \leftarrow cell\_num/node\_num$;
Calculate the node number of the last Cell-Graph;
for $i = 1,2,\dots graph\_num - 1$ **do**
    Load the cell coordinates;
    for $j = 1,2,\dots node\_num$ **do**
        for $k = j \dots node\_num$ **do**
            Calculate the link values of node pair of (j,k) and (k,j) according to Eq. (2);
            Attach the link values to the adjacent matrix;
            Attach the node index and edge index;
            Attach the node features;
        end for
    end for
end for
Calculate the node number of the last Cell-Graph: $last\_node\_num \leftarrow (cell\_num\%node\_num)$;
Load the cell coordinates;
for $j = 1,2,\dots last\_node\_num$ **do**
    for $k = j \dots last\_node\_num$ **do**
        Calculate the link values of node pair of (j,k) and (k,j) according to Eq. (2);
        Attach the link values to the adjacent matrix;
        Attach the node index and edge index;
        Attach the node features;
    end for
end for
Save all the Cell-Graphs.

GNN model architectures

When designing the GNN model architectures, we considered two different types of convolutional unit, including GCNConv and GINConv. The major difference between GCNConv and GINConv is reflected by the different mechanism of the message passing (i.e. node feature passing), as illustrated in Figure 2. More specifically, GCN[1] is the graph convolutional network, which calculates the node features by aggregating features of the node and its neighbors, as shown in Figure 2a. In contrast, GIN[2] is the graph isomorphism network, which adds an extra multilayer perceptron to generate the outputs, as shown in Figure 2b. The graph convolution needs to be combined with pooling layers. We tested the performance of models with two types of pooling layers: TopKPooling[3–5] and SAGPooling[5,6]. They provide an effective way to preserve the critical graph features and structures by using two different ways of calculating the projection matrix of node scores. TopKPooling calculates $y = \text{softmax}(Xp)$ with the trainable projection weight p, while SAGPooling uses a GNN to extract the ranking score for the nodes by $y = \text{softmax}(\text{GNN}(X,A))$.

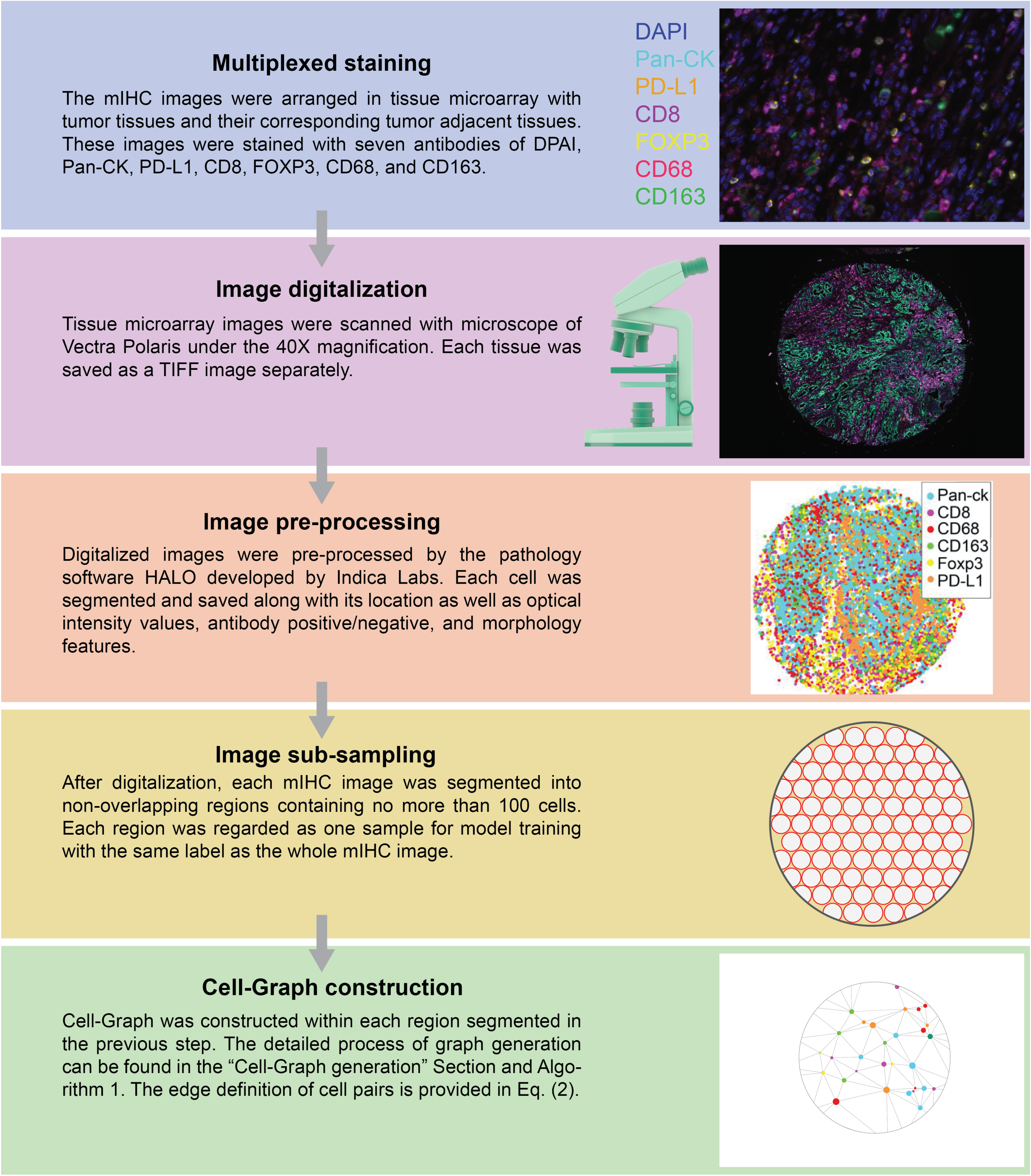

Supplementary Figure 1. An overview of the key procedures for multiplexed staining and image processing to generate Cell-Graphs. A detailed description of each step can be seen in the corresponding panel of the figure.

Metrics for evaluating the model performance

The model performance was evaluated with the following commonly used metrics and measures, including Accuracy (ACC), F1-score, Matthews Correlation Coefficient (MCC), and receiver-operating characteristic (ROC) curve with the corresponding

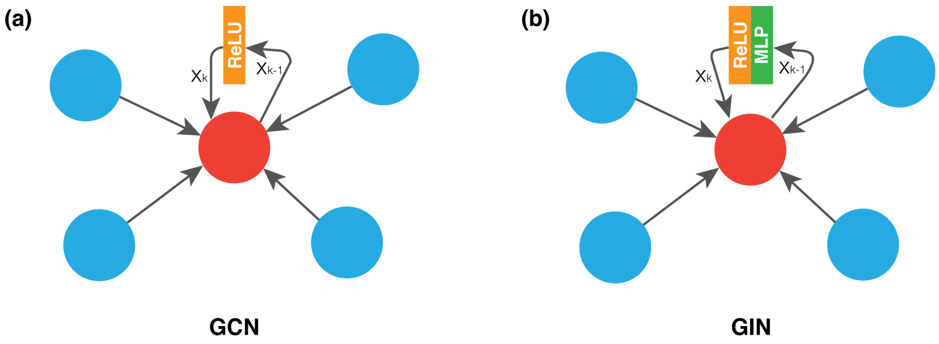

Supplementary Figure 2. An illustration of message passing operation of GCN and GIN. Here shows the source node's (Red) calculation using the node attributes of its neighbor nodes (Blue) and source node itself. **(a)** shows the message passing of GCN, while **(b)** displays the operation of GIN.

area under the ROC curve (AUROC). These performance metrics are defined by the following equations:

$$
\begin{aligned}
\text{Recall} &= \frac{TP}{TP+FN} \\
\text{Precision} &= \frac{TP}{TP+FP} \\
\text{ACC} &= \frac{TP+TN}{TP+TN+FP+FN} \\
\text{F1-Score} &= \frac{2 \times Recall \times Precision}{Recall + Precision} \\
\text{MCC} &= \frac{(TP \times TN) + (FP \times FN)}{\sqrt{(TP+FP)(TP+FN)(TN+FP)(TN+FN)}}.
\end{aligned}
$$

Here, *TP* represents true positive; *TN*, true negative; *FP*, false positive; *FN*, false negative. For all the measures defined above, a higher value indicates a better performance of the model. The corresponding AUC values are calculated as the primary performance metric to evaluate the performance of the trained models and compare between different methods.

Strategies to prevent overfitting

Due to the huge size of cancer histopathology images, segmentation of the images at the patch- or tile-levels prior to deep learning-based model training is a common practice in digital pathology. To avoid the overfitting issue, in this study we applied multiple strategies: 1) use of a strict early stopping strategy; 2) dynamic learning rate, and 3) use of pooling layers, to effectively avoid the model overfitting. Moreover, the training and validation loss value changes also provide useful clues in regards to whether or not the trained deep learning model was subjected to overfitting. Figures 10-19 show the detailed training and validation loss value changes for binary- and ternary-classification model training on five-fold cross-validation. The position of the early stopping was indicated with the red dash line in each figure. As can be observed in these figures, all the models was stopped when the validation loss stopped further decreasing with the patience of 20 epochs. For most models, they required training for 30 to 80 epochs to achieve the optimal. Due to the adopted multiple strategies and results, our models were unlikely to get overfitted.

Supplementary tables and figures

Supplementary Table 1. Clinical characteristics of the gastric cancer patient cohort used in this study. The patient numbers of different gender, survival status, TNM stages, and their survival time interval are included. Two data-binning strategies were applied to segment patients into binary- and ternary-class.

Variables		Number of patients	Remarks
Gender	Male	124	1 unknown
	Female	47	
Status	Deceased	113	
	Alive	59	
TNM Stage (the AJCC 8th edition)	Stage I	14	8 unknown
	Stage II	52	
	Stage III	95	
	Stage IV	3	
Overall survival	$\leq$ 1 yr	51	Ternary-class dataset
	>1 and $\leq$ 5 yrs	60	
	>5 yrs	61	
	$\leq$ 2 yrs	82	Binary-class dataset
	$\geq$ 4 yrs	70	

Supplementary Table 2. Clinical characteristics of the holdout cohorts for independent test and survival analysis. The class balance was considered in the data split, and the patient numbers of different gender, survival status, TNM stage, and their survival time interval are shown in the table.

	Binary-class testing cohort		Ternary-class testing cohort	
	Variables	Number of patients	Variables	Number of patients
Gender	Male	40	Male	28
	Female	12	Female	7
Status	Deceased	38	Deceased	22
	Alive	14	Alive	13
TNM Stage (the AJCC 8th edition)	Stage I	5	Stage I	3
	Stage II	12	Stage II	14
	Stage III	31	Stage III	16
	Stage IV	0	Stage IV	1
Overall survival	$\leq$ 2 yr	17	$\leq$ 1 yr	8
	>2 and $\leq$ 4 yrs	20	>1 and $\leq$ 5 yrs	14
	>4 yrs	14	>5 yrs	13

Supplementary Table 3. Low-pass wavelet decomposition of short-term, medium-term, and long-term samples with features of Pan-CK and Cell Area. The decomposition was conducted on one low-pass channel and two high-pass channels. In each figure, the decomposition coefficients were visualized by gradient color from red to blue, where red represents the high-valued coefficient and blue represents low-valued coefficient. The low-pass composition of features of Pan-CK and Cell Area is shown. No significant differences of short-term, medium-term, and long-term patients can be observed from the following figures.

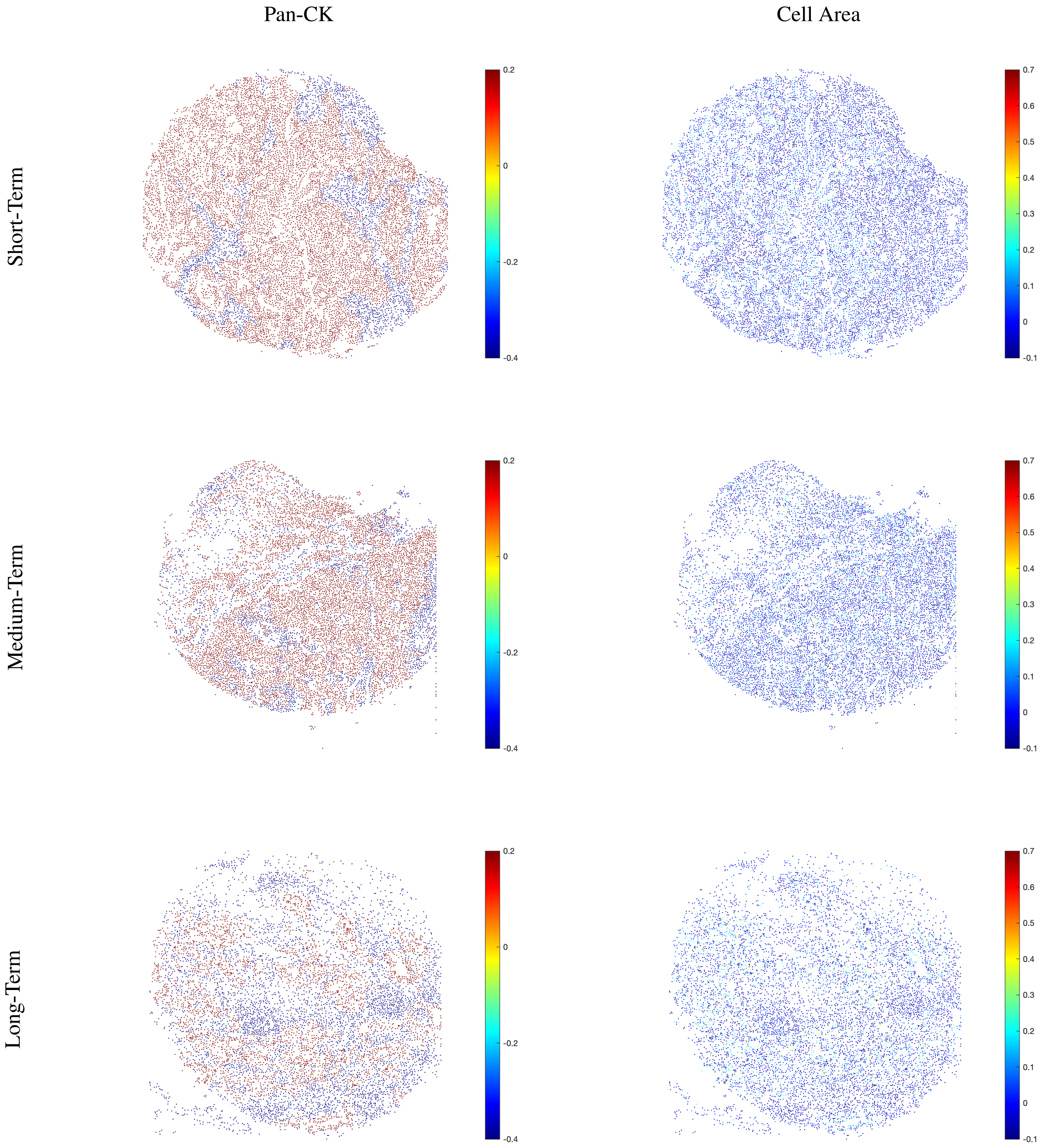

Supplementary Table 4. Low-pass wavelet decomposition of short-term, medium-term, and long-term samples with features of Cytoplasm Area and Nucleus Area. The decomposition was conducted on one low-pass channel and two high-pass channels. In each figure, the decomposition coefficients were visualized by gradient color from red to blue, where red represents the high coefficient and blue represents low coefficient. Here shows the low-pass composition on features of Cytoplasm Area and Nucleus Area, no significant differences of short-term, medium-term, and long-term patients can be observed from the figures in this table.

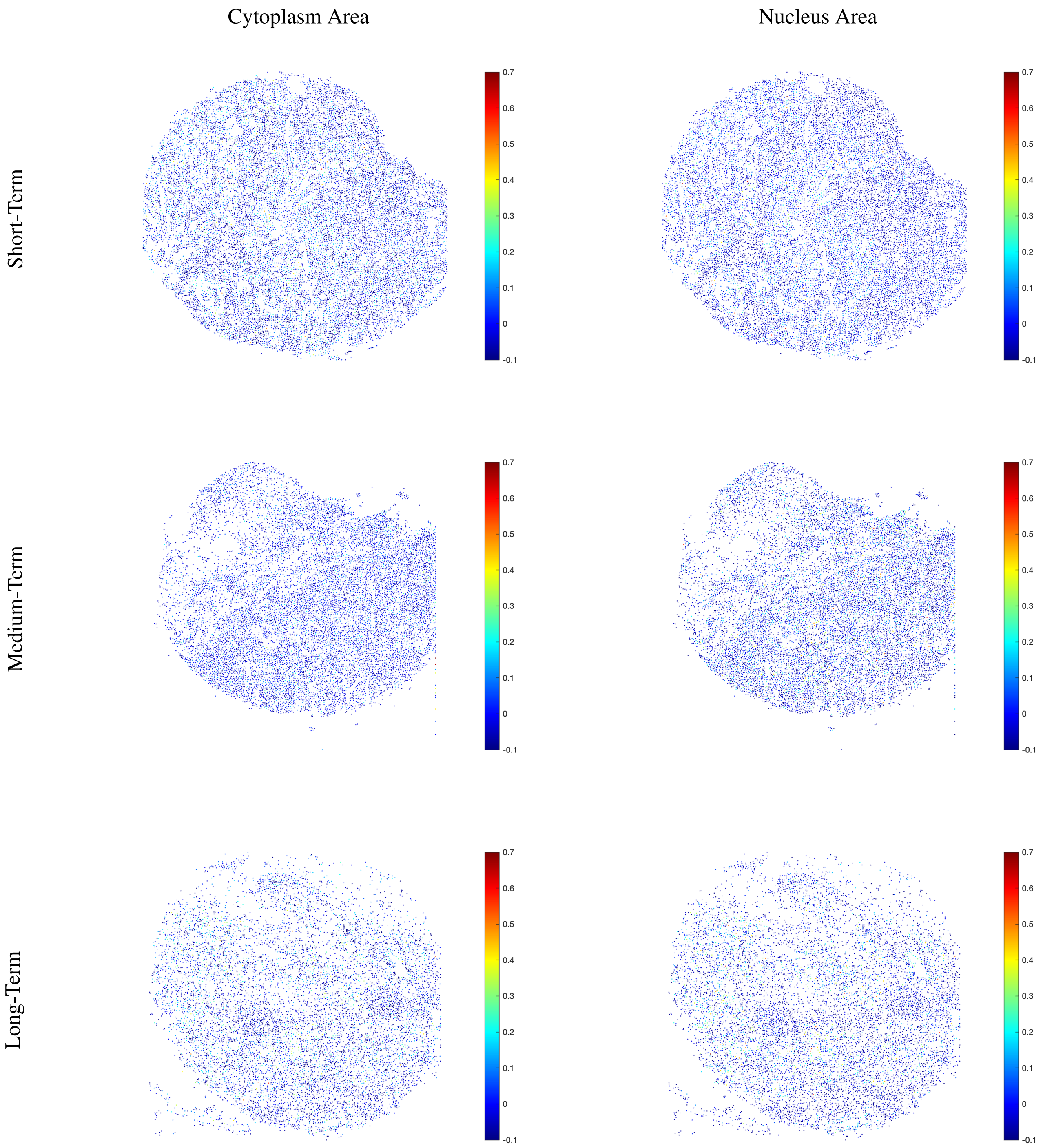

Supplementary Table 5. Low-pass wavelet decomposition of short-term, medium-term, and long-term samples with features of Nucleus Perimeter and Nucleus Roundness. The decomposition was conducted on one low-pass channel and two high-pass channels. In each figure, the decomposition coefficients were visualized by gradient color from red to blue, where red represents the high coefficient and blue represents low coefficient. Here shows the low-pass composition on features of Nucleus Perimeter and Nucleus Roundness, no significant differences of short-term, medium-term, and long-term patients can be observed from the following figures.

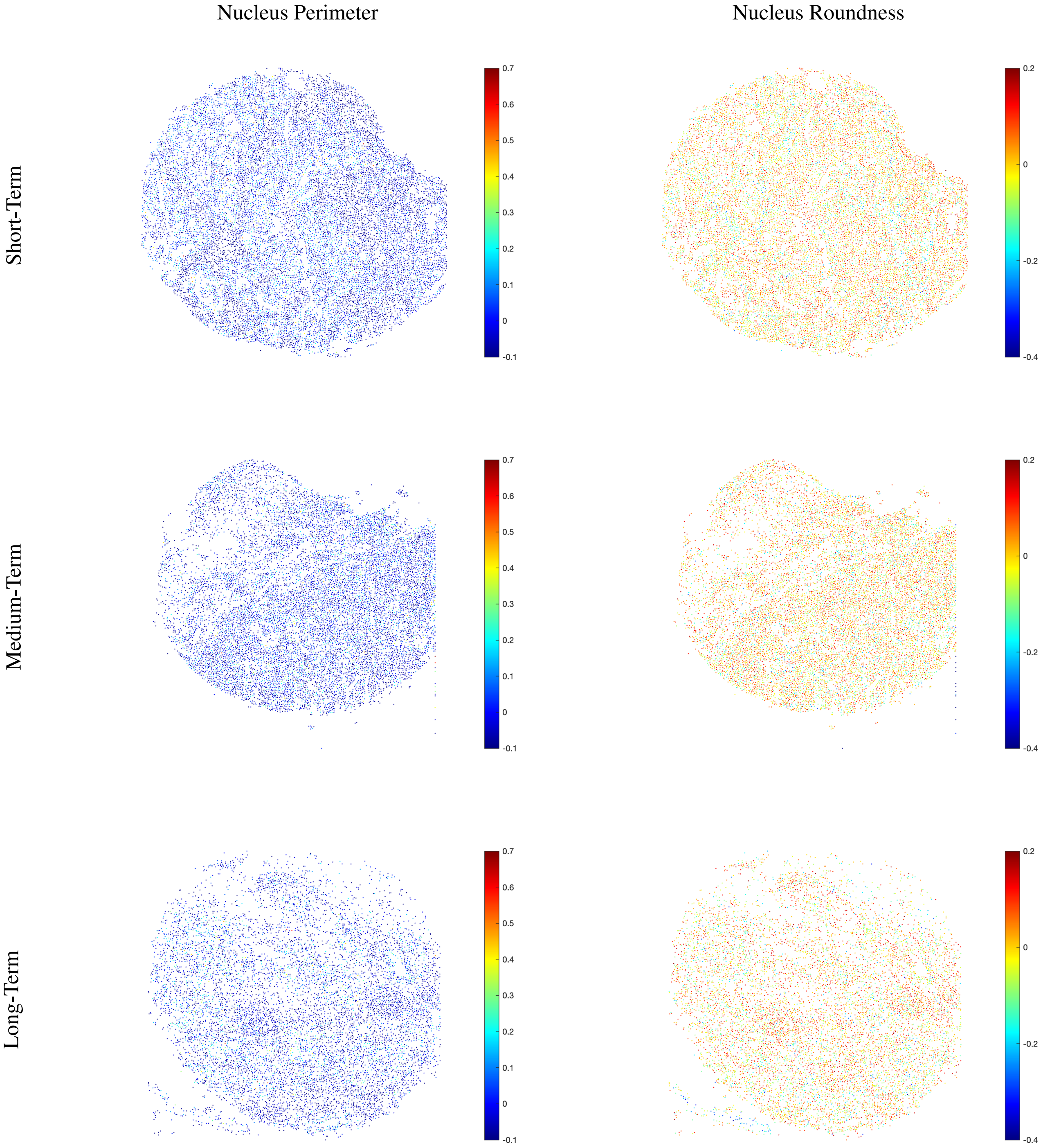

Supplementary Table 6. High-pass (channel 1) wavelet decomposition of short-term, medium-term, and long-term samples with features of Pan-CK and Cell Area. The decomposition was conducted on one low-pass channel and two high-pass channels. In each figure, the decomposition coefficients were visualized by gradient color from red to blue, where red represents the high coefficient and blue represents low coefficient. The figures below show the high-pass (channel 1) composition of features of Pan-CK and Cell Area. Major color differences of short-term (dominated by red color), medium-term (red and blue mixed), and long-term (dominated by blue) patients can be observed from the figures in the Table.

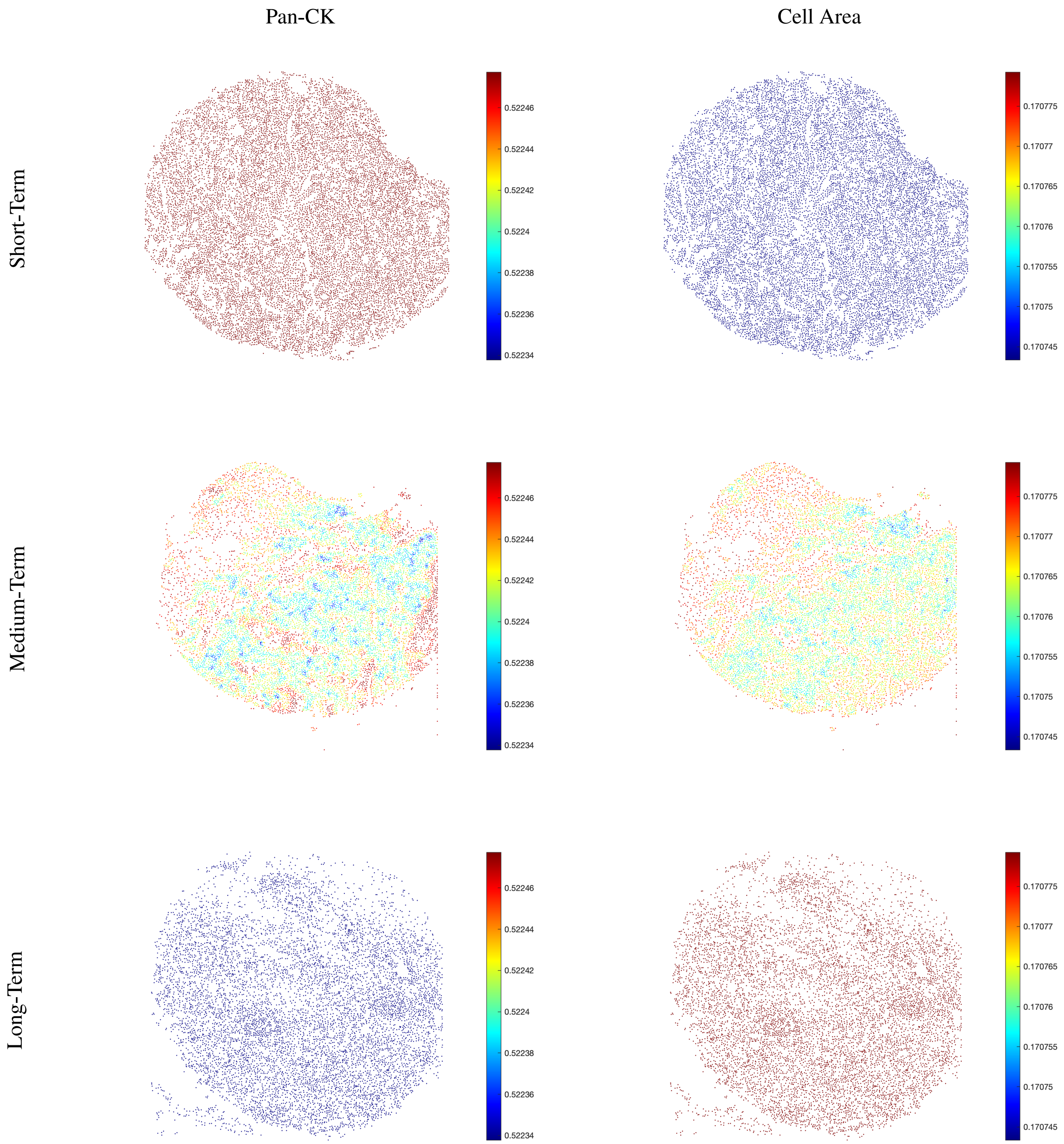

Supplementary Table 7. High-pass (channel 1) wavelet decomposition of short-term, medium-term, and long-term samples with features of Cytoplasm Area and Nucleus Area. The decomposition was conducted on one low-pass channel and two high-pass channels. In each figure, the decomposition coefficients were visualized by gradient color from red to blue, where red represents the high coefficient and blue represents low coefficient. Here shows the high-pass (channel 1) composition on features of Cytoplasm Area and Nucleus Area, major color differences of medium-term (mixed color of red and green) from short- and long-term (dominated by single color of red or blue) patients can be observed from the following figures.

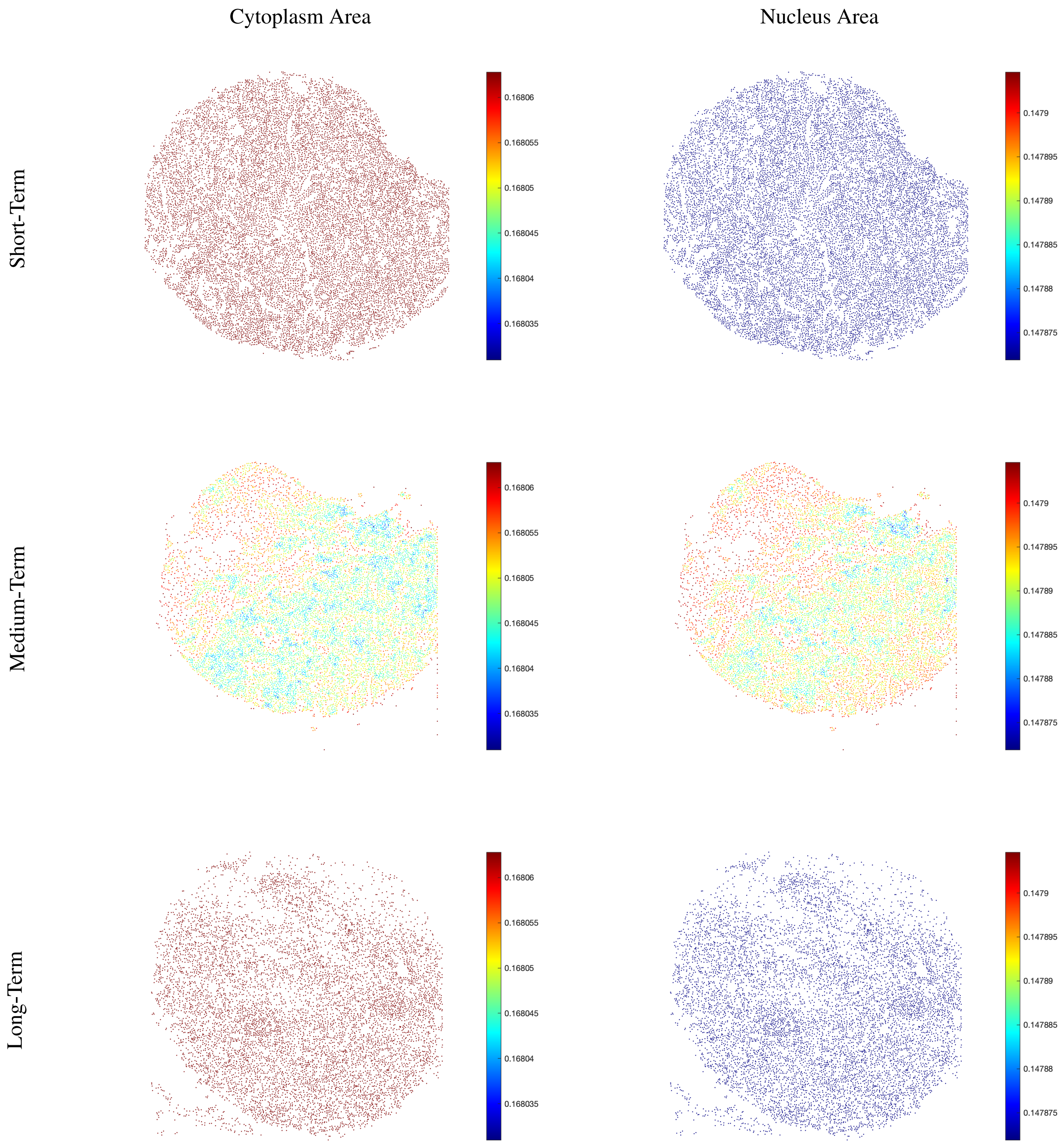

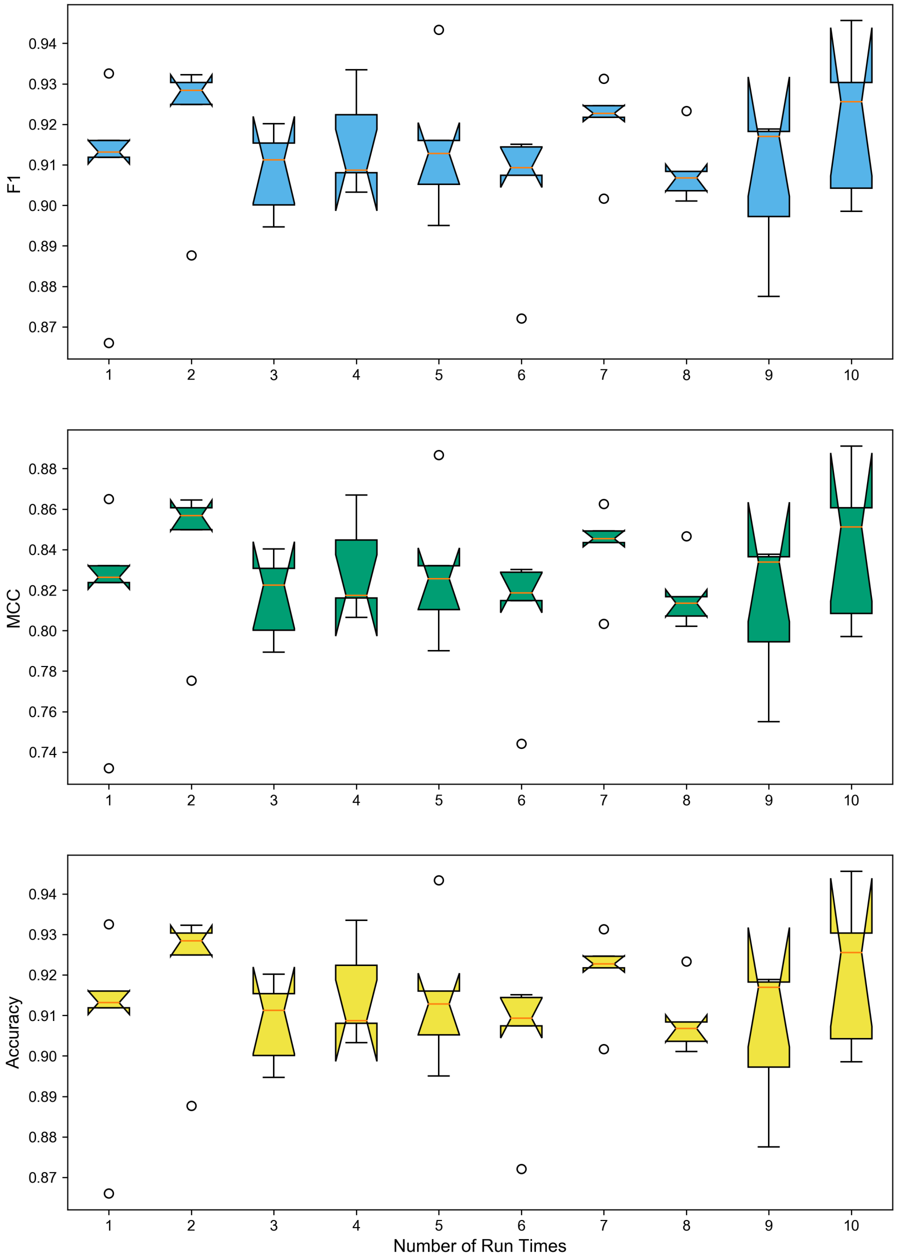

Supplementary Figure 3. Model performance on ten repetitions of five-fold cross-validation on binary classification using GINTopK. To further confirm the stability of the proposed model architecture, the ten repetitions of five-fold cross-validation were conducted with randomly initialized weights for each training. According to the figure, the median values of both Accuracy and F1-score were within the range of 0.90-0.93 (MCC values ranged from 0.80 to 0.86), thereby suggesting the stability of our proposed GINTopK model.

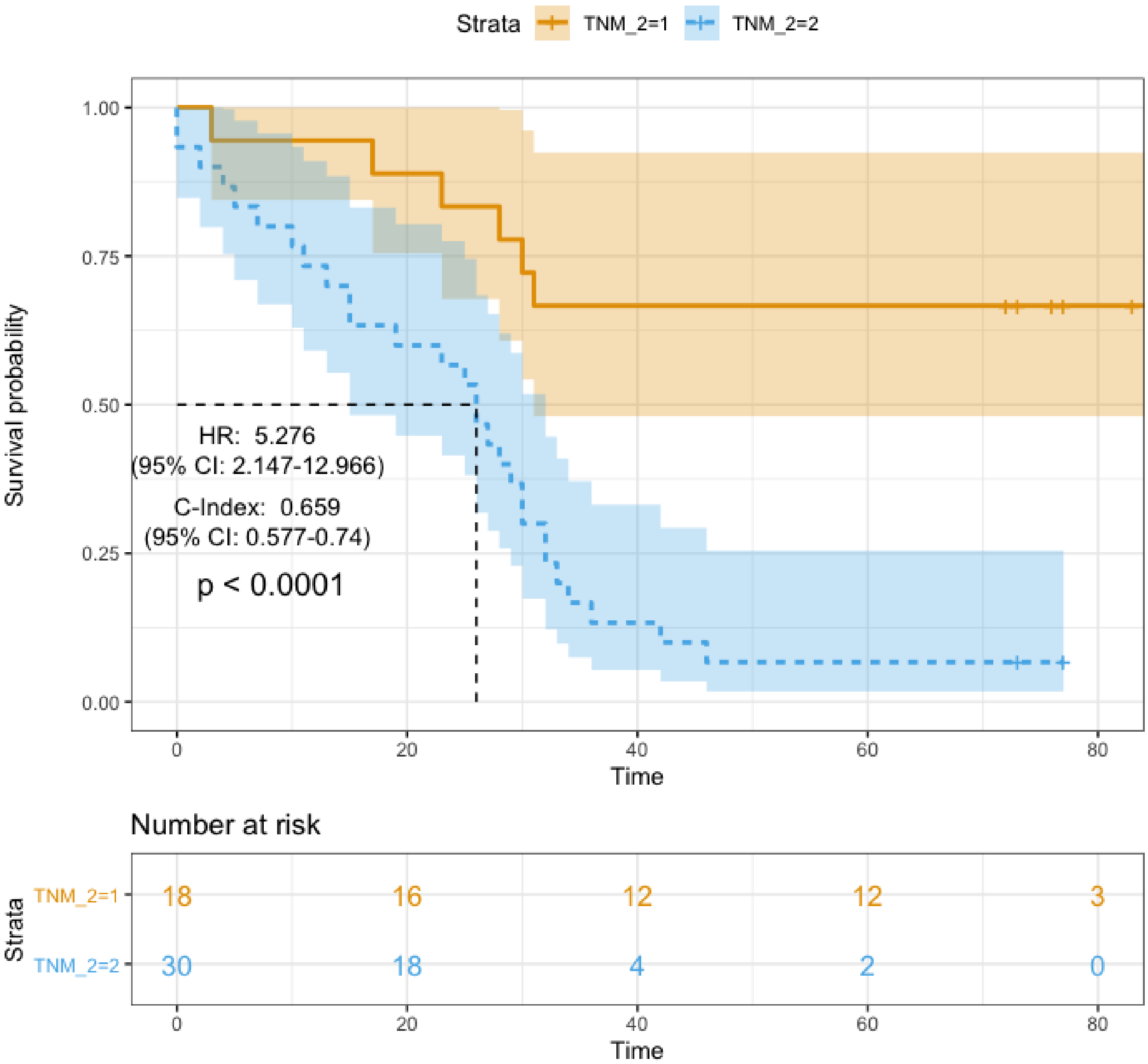

Supplementary Figure 4. Kaplan-Meier survival analysis of patient overall survival based on the TNM-2 on binary testing cohort. As can be observed, Kaplan-Meier survival analysis shows that the TNM-2 (*I*, *II* vs. *III*) can stratify the patients into groups of low- and high-risk with C-Index (0.659 (95% CI: 0.577 − 0.740)), Hazard Ratio (5.276 (95% CI: 2.147 − 12.966)), and the *P-value* < 0.0001.

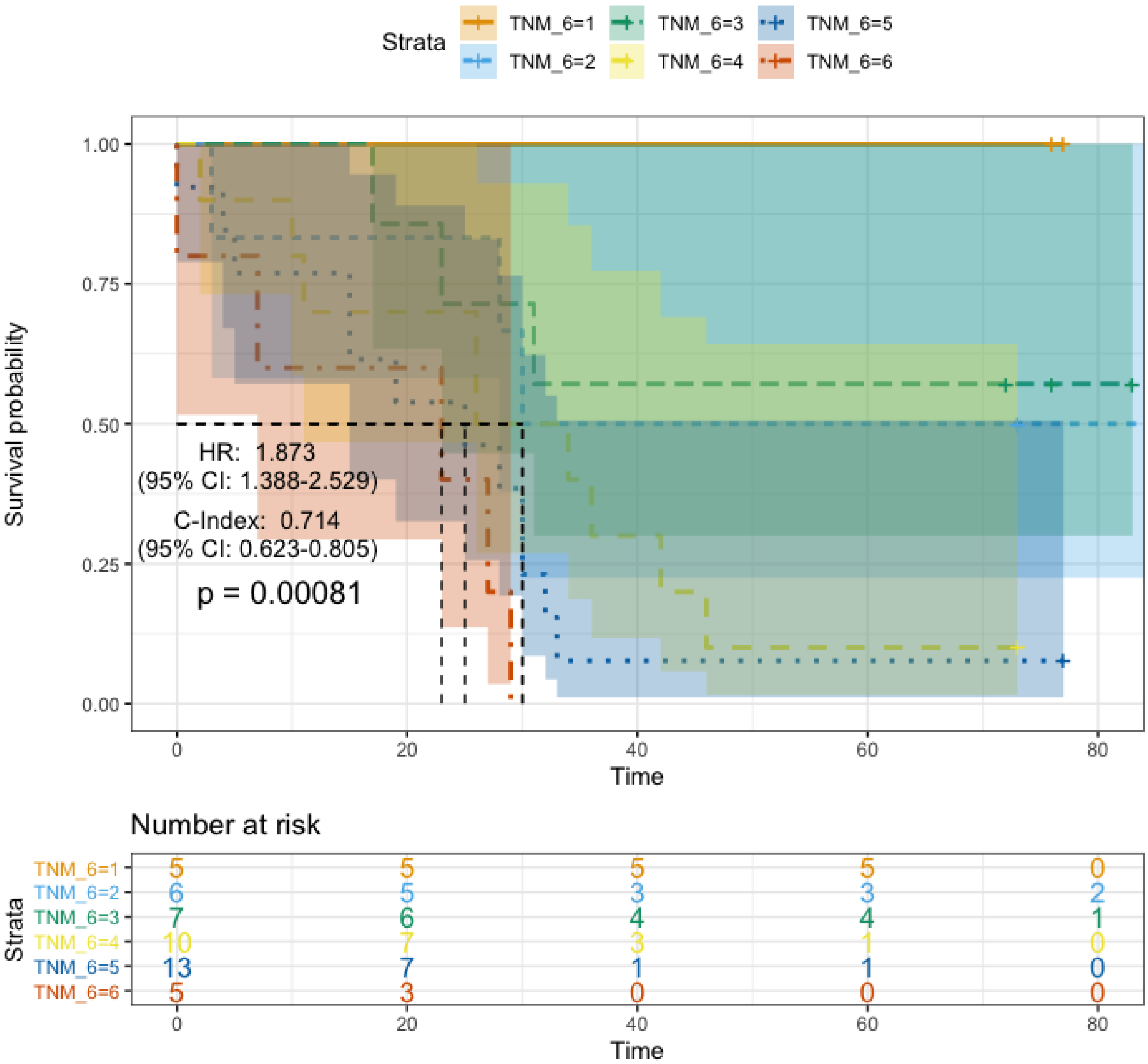

Supplementary Figure 5. Kaplan-Meier survival analysis of patient overall survival based on the TNM-6 on binary testing cohort. As can be observed, Kaplan-Meier survival analysis shows that the TNM-6 (I vs. II_A vs. II_B vs. III_A vs. III_B vs. III_C) can stratify the patients into six groups of different prognosis with C-Index (0.714 (95% CI: 0.623 − 0.805)), Hazard Ratio (1.873 (95% CI: 1.388 − 2.529)), and the *P-value* = 0.00081.

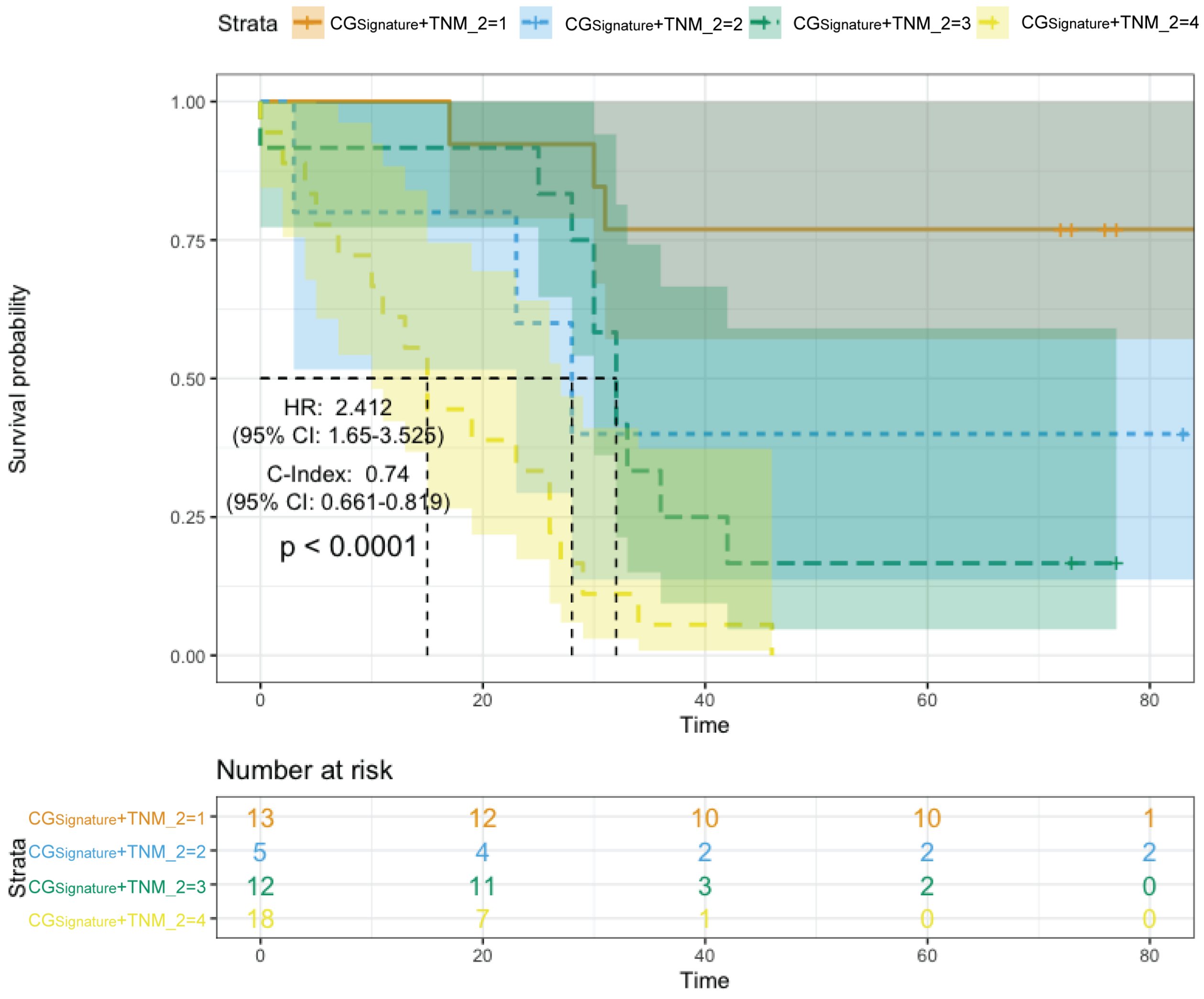

Supplementary Figure 6. Kaplan-Meier survival analysis of patient overall survival based on the combination of $CG_{Signature}$ and TNM-2 on binary testing cohort. As can be seen from the figure, Kaplan-Meier survival analysis shows that the combination of $CG_{Signature}$ and TNM-2 can stratify the patients into four groups of different prognosis with C-Index (0.740 (95% CI: 0.661 − 0.819)), Hazard Ratio (2.412 (95% CI: 1.650 − 3.525)), and the *P-value* < 0.0001.

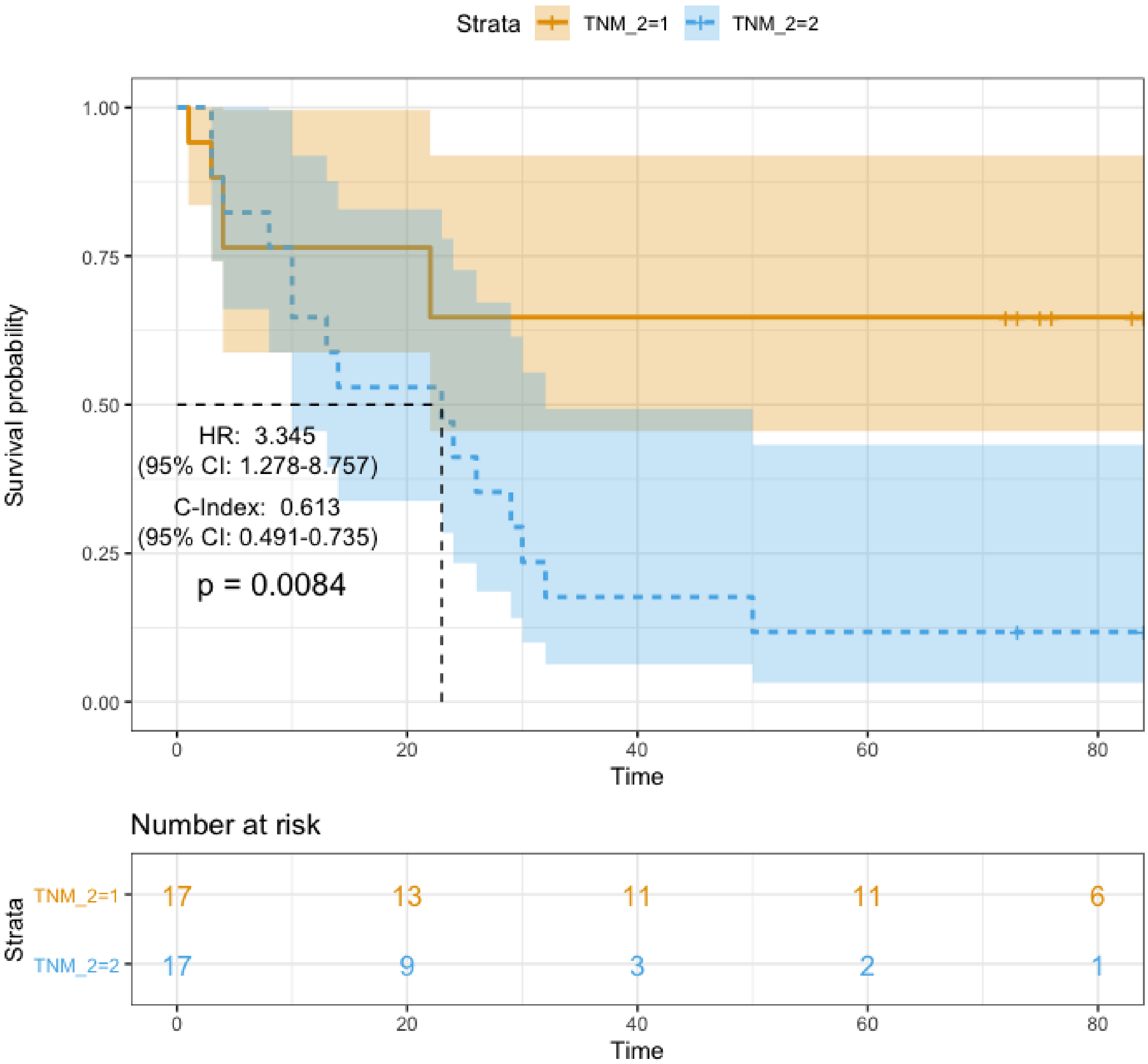

Supplementary Figure 7. Kaplan-Meier survival analysis of patient overall survival based on the TNM-2 on ternary testing cohort. As can be seen from the figure, Kaplan-Meier survival analysis shows that the TNM-2 (*I*, *II* vs. *III*) can stratify the patients into groups of low- and high-risk with C-Index (0.613 (95% CI: 0.491 − 0.735)), Hazard Ratio (3.345 (95% CI: 1.278 − 8.757)), and the *P-value* = 0.0084.

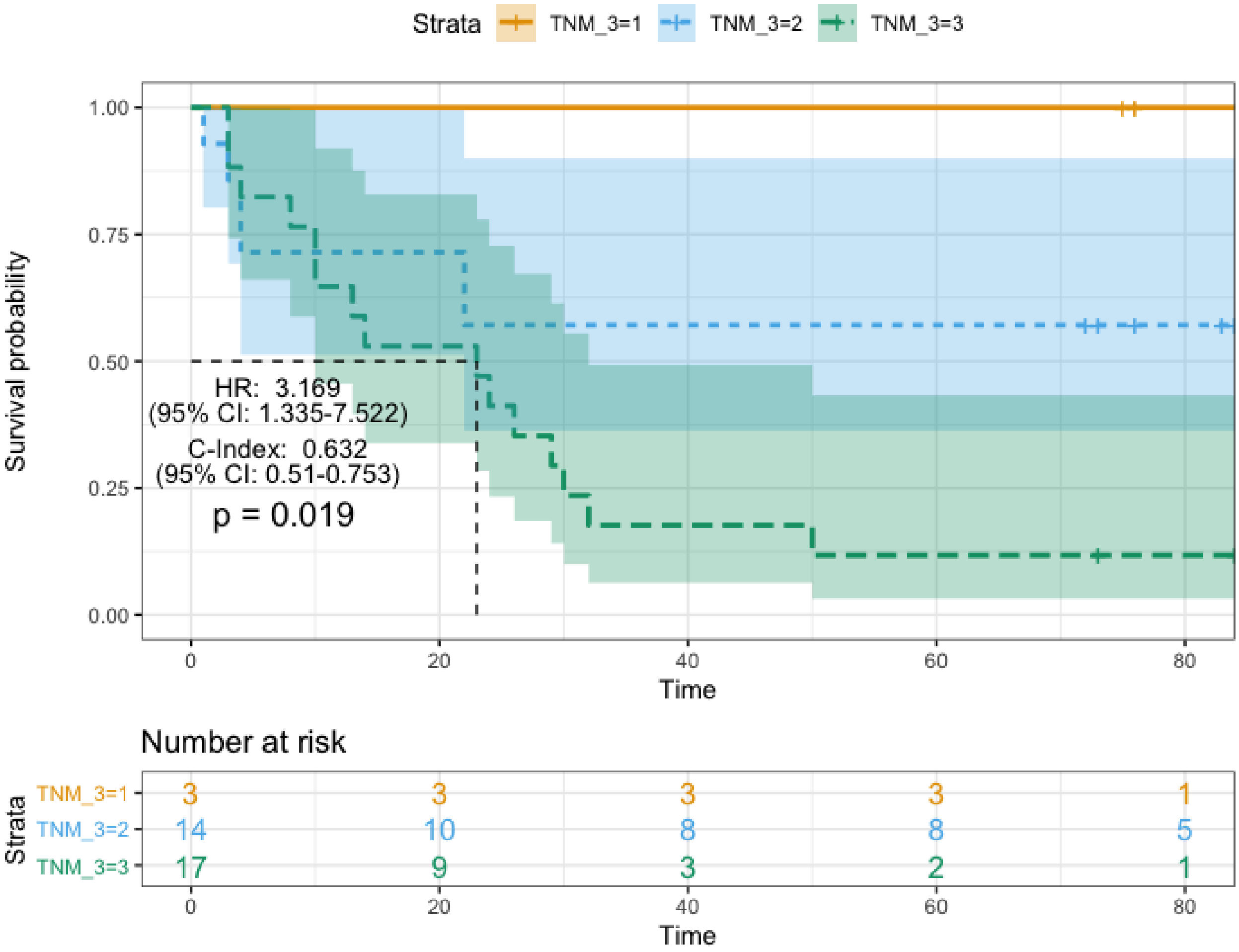

Supplementary Figure 8. Kaplan-Meier survival analysis of patient overall survival based on the TNM-3 on ternary testing cohort. As can be seen from the figure, Kaplan-Meier survival analysis shows that the TNM-3 (*I* vs. *II* vs. *III*) can stratify the patients into groups of low-, medium-, and high-risk with C-Index (0.632 (95% CI: 0.510 − 0.753)), Hazard Ratio (3.169 (95% CI: 1.335 − 7.522)), and the *P-value* = 0.019.

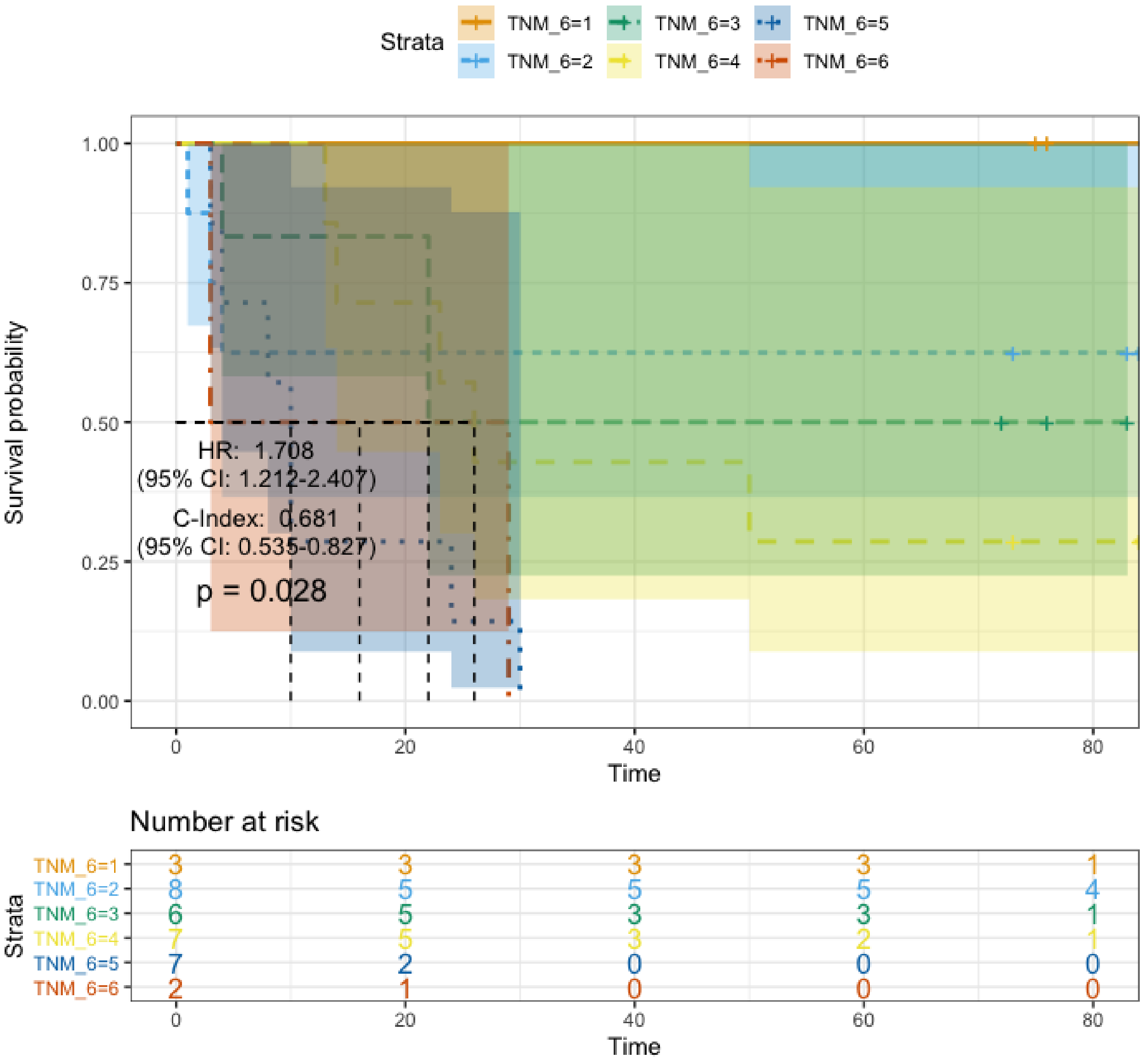

Supplementary Figure 9. Kaplan-Meier survival analysis of patient overall survival based on the TNM-6 on ternary testing cohort. As can be seen from the figure, Kaplan-Meier survival analysis shows that the TNM-6 (I vs. II_A vs. II_B vs. III_A vs. III_B vs. III_C) can stratify the patients into six groups of different prognosis with C-Index (0.681 (95% CI: $0.535 - 0.827$)), Hazard Ratio (1.708 (95% CI: $1.212 - 2.407$)), and the P-$value$ = 0.028.

Supplementary Table 8. High-pass (channel 1) wavelet decomposition of short-term, medium-term, and long-term samples with features of Nucleus Perimeter and Nucleus Roundness. The decomposition was conducted on one low-pass channel and two high-pass channels. In each figure, the decomposition coefficients were visualized by gradient color from red to blue, where red represents the high coefficient and blue represents low coefficient. Here shows the high-pass (channel 1) composition on features of Nucleus Perimeter and Nucleus Roundness. For feature Nucleus Perimeter, major color differences of short- (dominated by blue color), medium- (mixed color of red and green) and long-term (dominated by red color) patients can be observed, while only differences of medium-term (mixed color of red and green) from the patients of the other two classes (dominated by red color) can be seen on the decomposition of Nucleus Roundness.

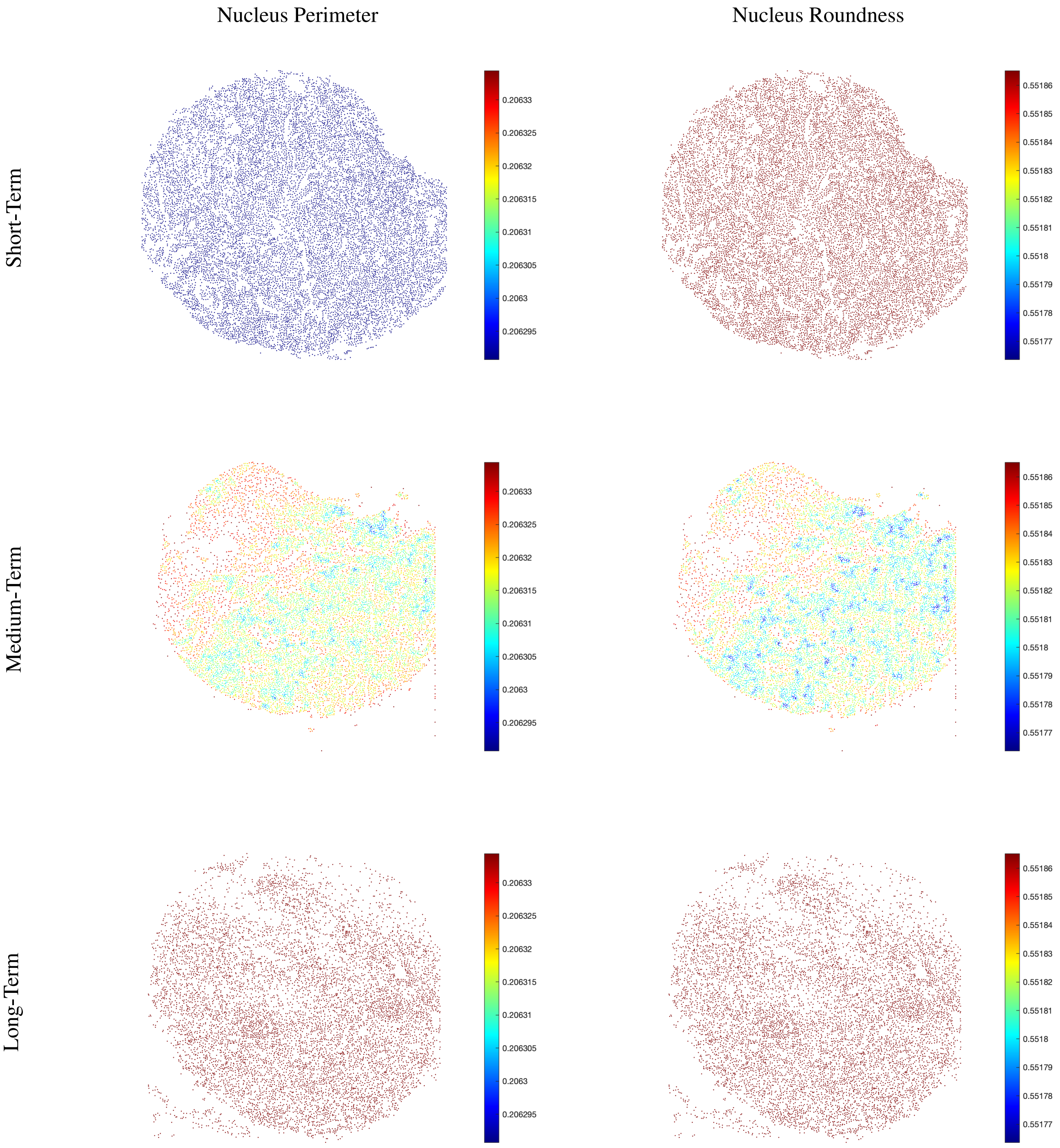

Supplementary Table 9. High-pass (channel 2) wavelet decomposition of short-term, medium-term, and long-term samples with features of Pan-CK and Cell Area. The decomposition was conducted on one low-pass channel and two high-pass channels. In each figure, the decomposition coefficients were visualized by gradient color from red to blue, where red represents the high coefficient and blue represents low coefficient. Here shows the high-pass (channel 2) composition on features of Pan-CK and Cell Area, major color differences of short-term (dominated by single color of red or blue), medium-term (mixed color from red to blue), and long-term (dominated by single color of blue or red) patients can be observed from the figures.

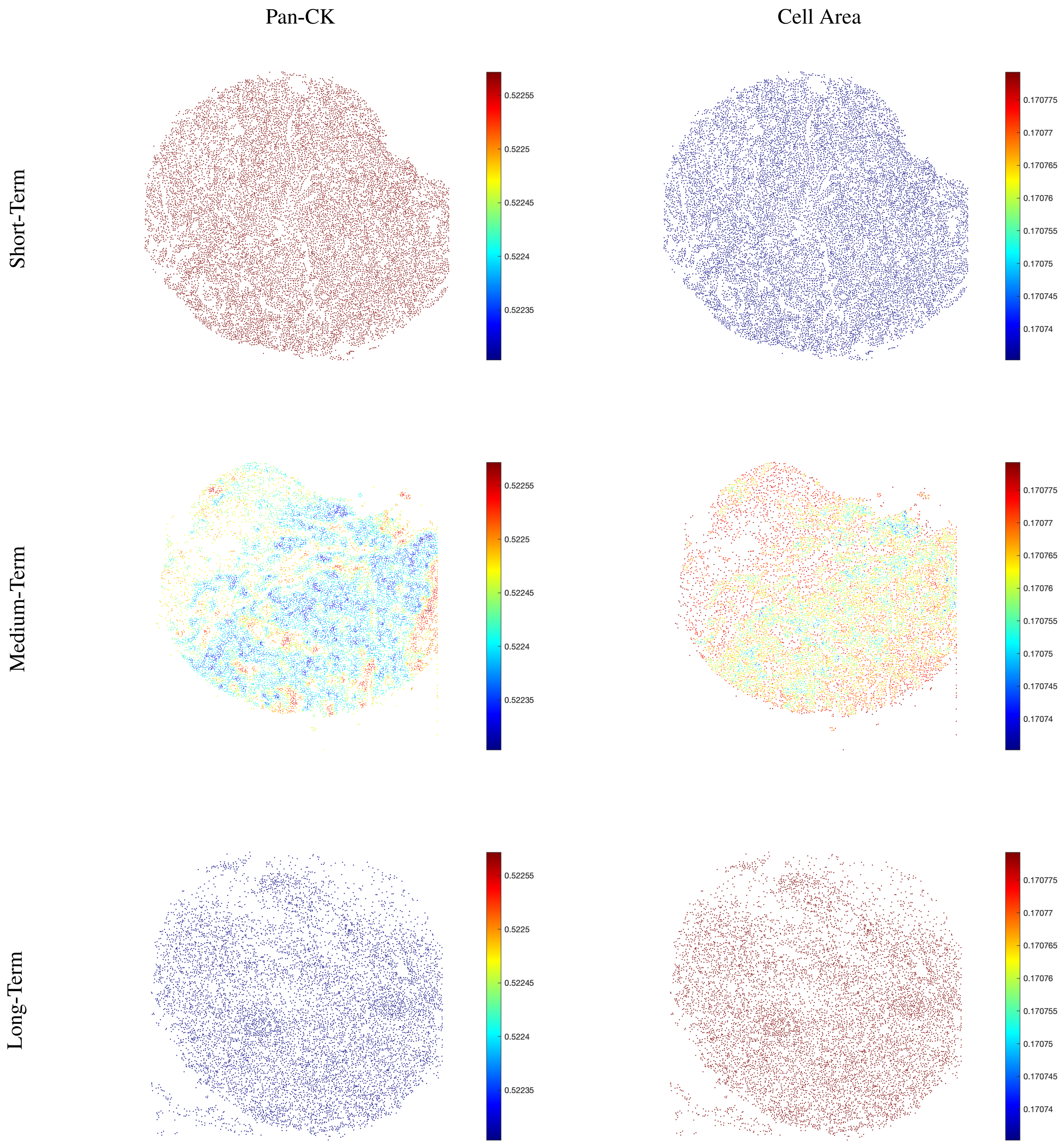

Supplementary Table 10. High-pass (channel 2) wavelet decomposition of short-term, medium-term, and long-term samples with features of Cytoplasm Area and Nucleus Area. The decomposition was conducted on one low-pass channel and two high-pass channels. In each figure, the decomposition coefficients were visualized by gradient color from red to blue, where red represents the high coefficient and blue represents low coefficient. Here shows the high-pass (channel 2) composition on features of Cytoplasm Area and Nucleus Area, major color differences of medium-term (mixed color of red and green) patient from short- and long-term (dominated by single color of red or blue) patients can be observed from following figures.

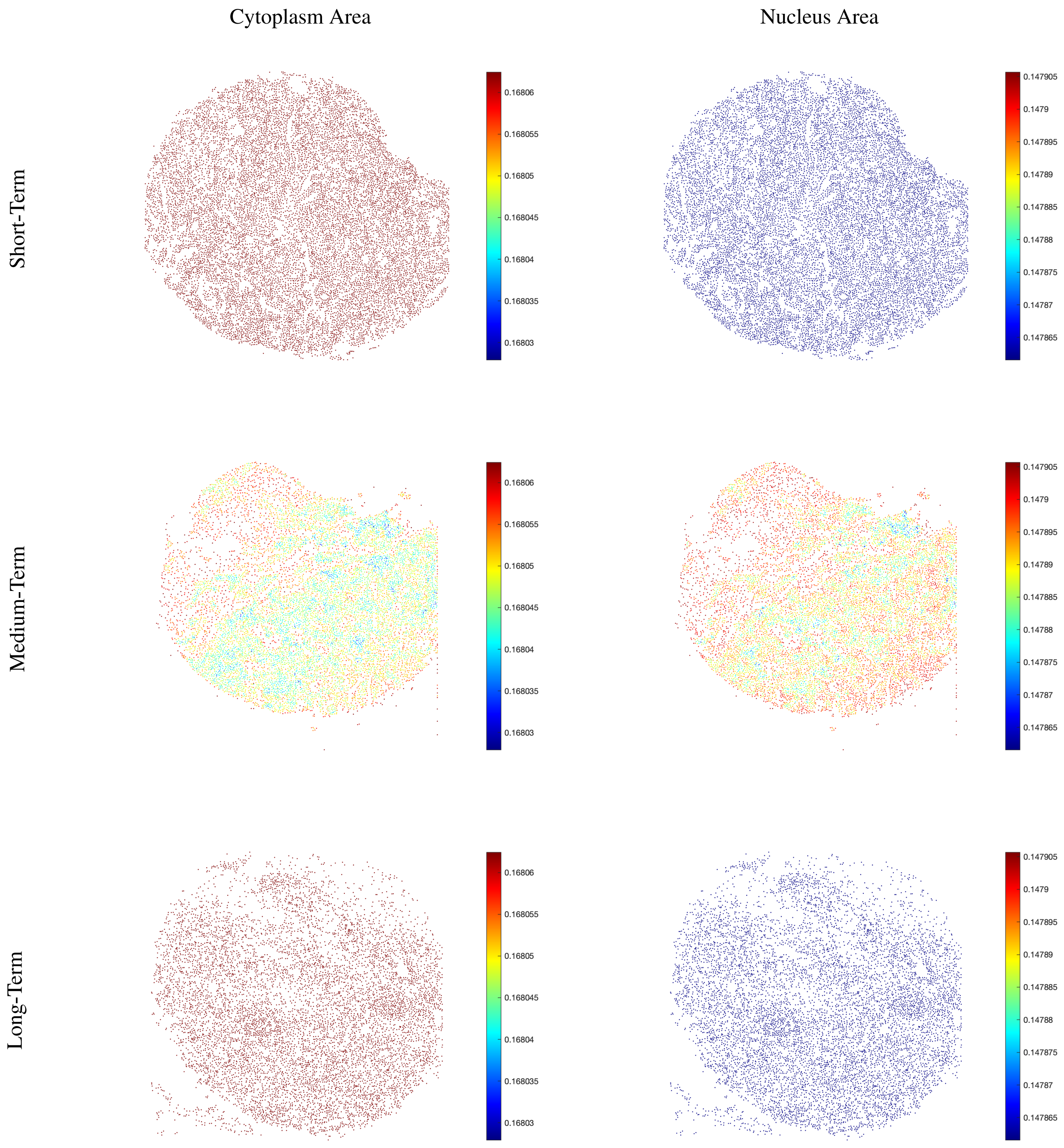

Supplementary Table 11. High-pass (channel 2) wavelet decomposition of short-term, medium-term, and long-term samples with features of Nucleus Perimeter and Nucleus Roundness. The decomposition was conducted on one low-pass channel and two high-pass channels. In each figure, the decomposition coefficients were visualized by gradient color from red to blue, where red represents the high coefficient and blue represents low coefficient. Here shows the high-pass (channel 2) composition on features of Nucleus Perimeter and Nucleus Roundness. For feature Nucleus Perimeter, major color differences of short- (dominated by blue color), medium- (mixed color of red and green) and long-term (dominated by red color) patients can be observed, while only differences of medium-term (mixed color of red and blue) from the patients of the other two classes (dominated by red color) can be seen on the decomposition of Nucleus Roundness.

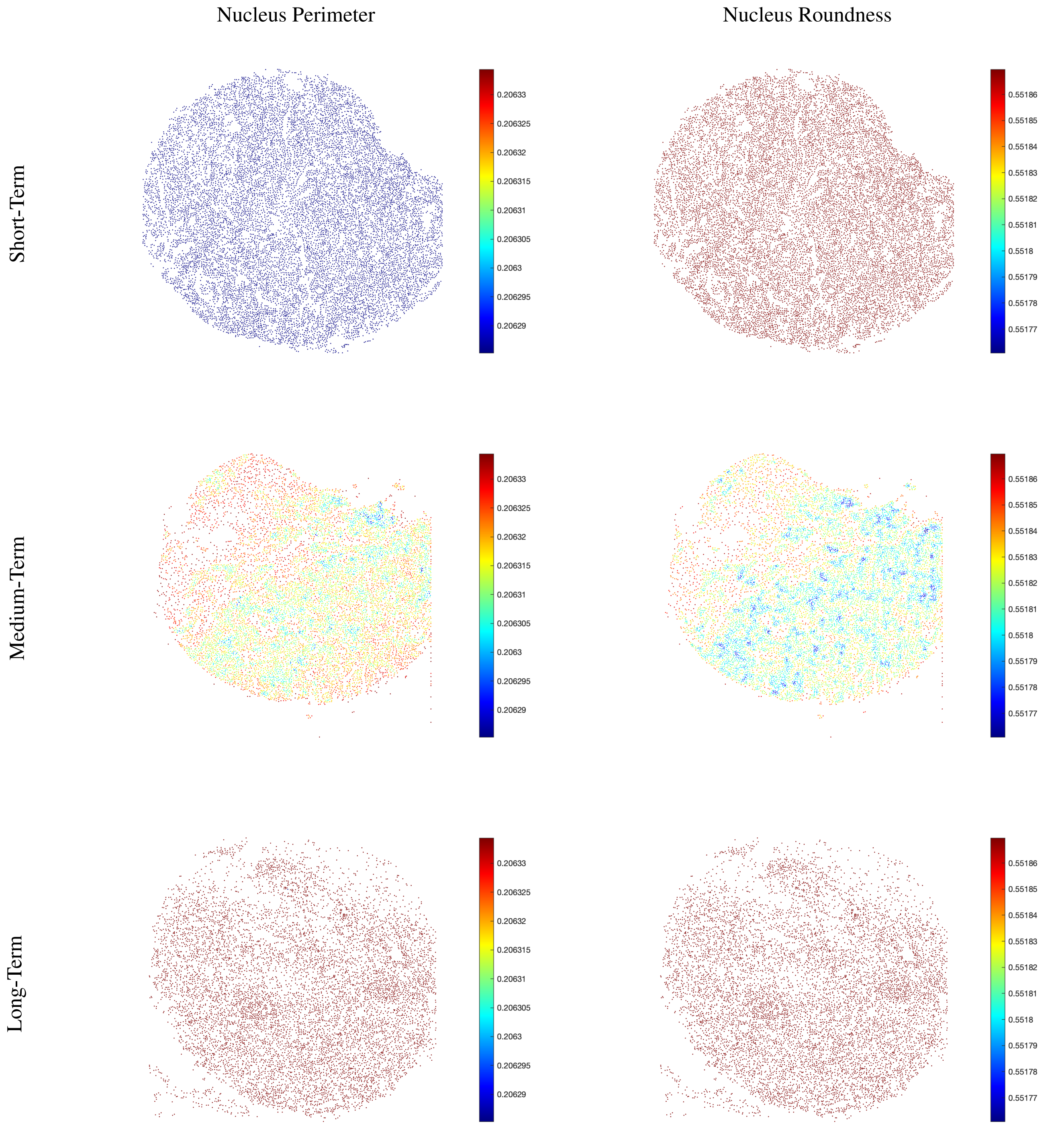

Supplementary Table 12. Information of antibodies used in this study.

Primary Ab	Dilution	Company	Catalogue number	Secondary Ab	Dyes
CD68	1:1200	Panovue	PPA101A200025	HRP	540
CD8a	1:300	CST	78701T	HRP	620
Pan-Keratin	1:200	CST	4545	HRP	690
Foxp3 (D2W8E™)	1:100	CST	98377S	HRP	570
CD163	1:100	Panovue	PPA102A20025	HRP	650
PD-L1	1:500	CST	78701T	HRP	520

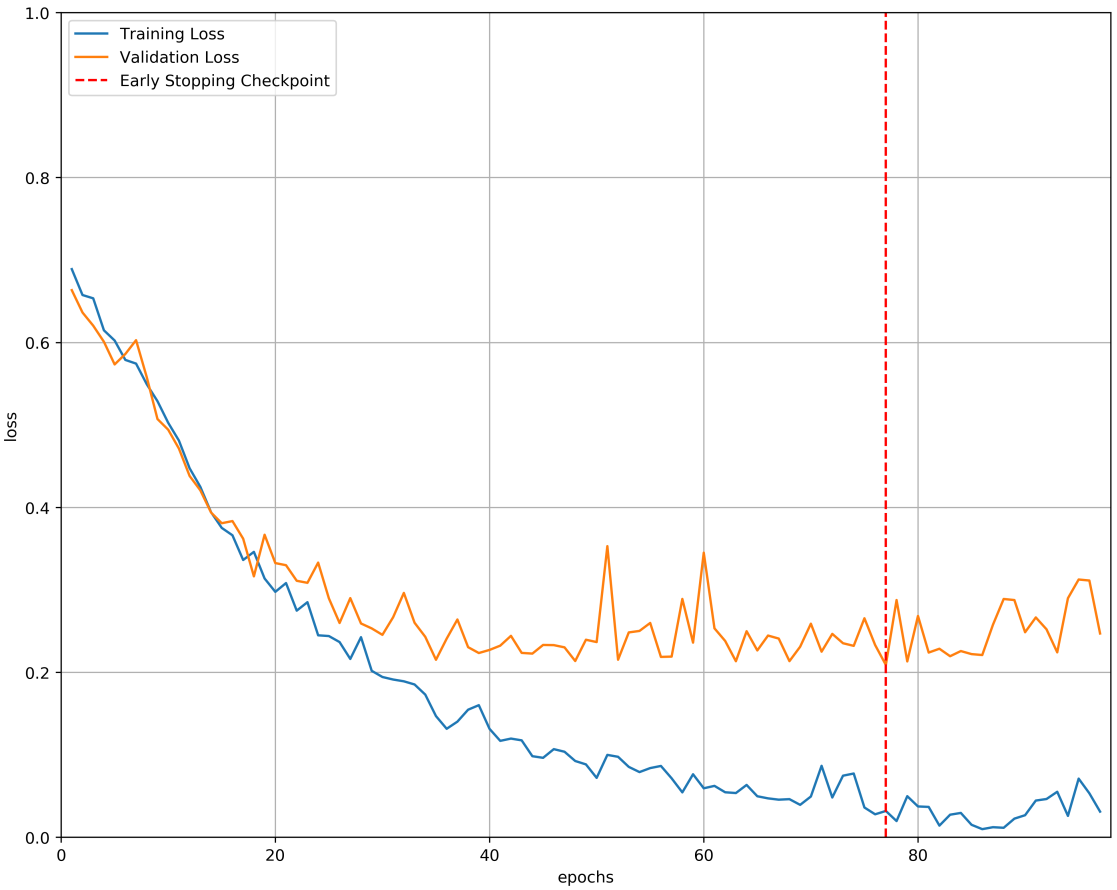

Supplementary Figure 10. Training and validation loss value changing along with the binary-class model training on Fold-0 subset. The position marked by the red dash line is the checkpoint of the final optimal model.

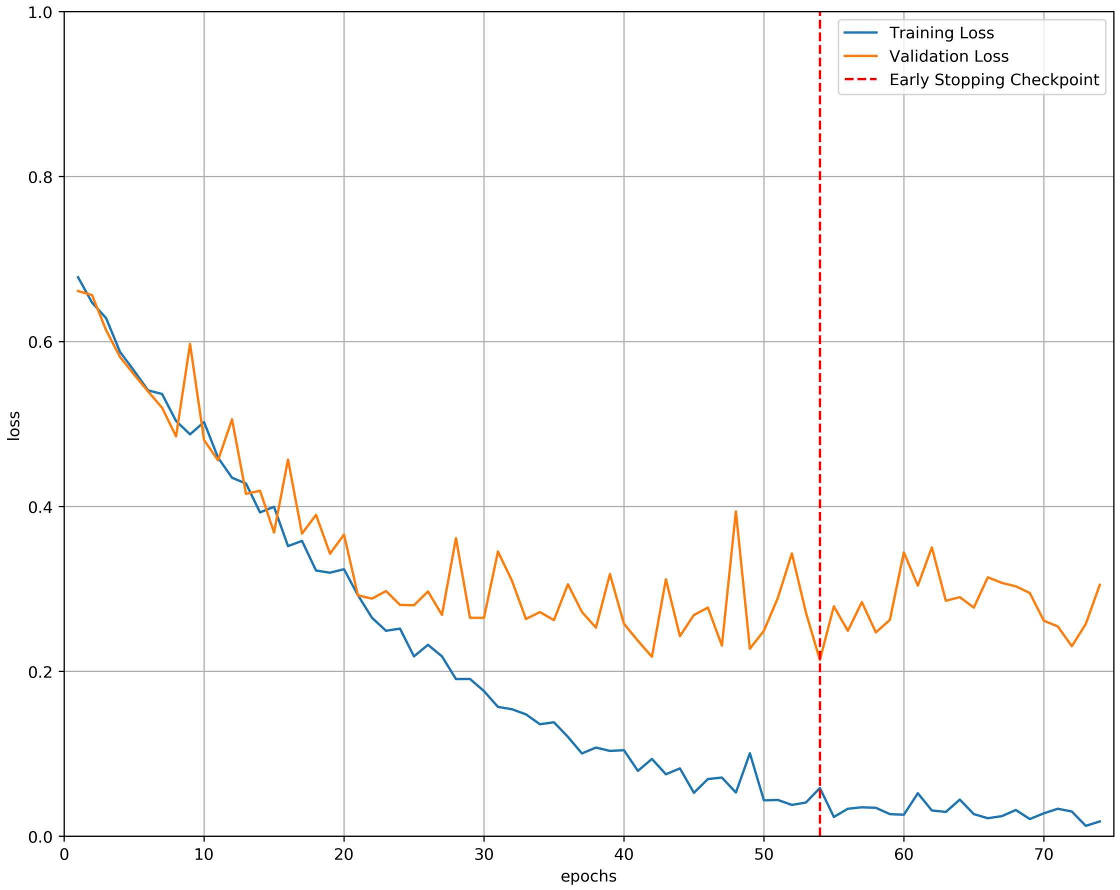

Supplementary Figure 11. Training and validation loss value changing along with the binary-class model training on Fold-1 subset. The position marked by the red dash line is the checkpoint of the final optimal model.

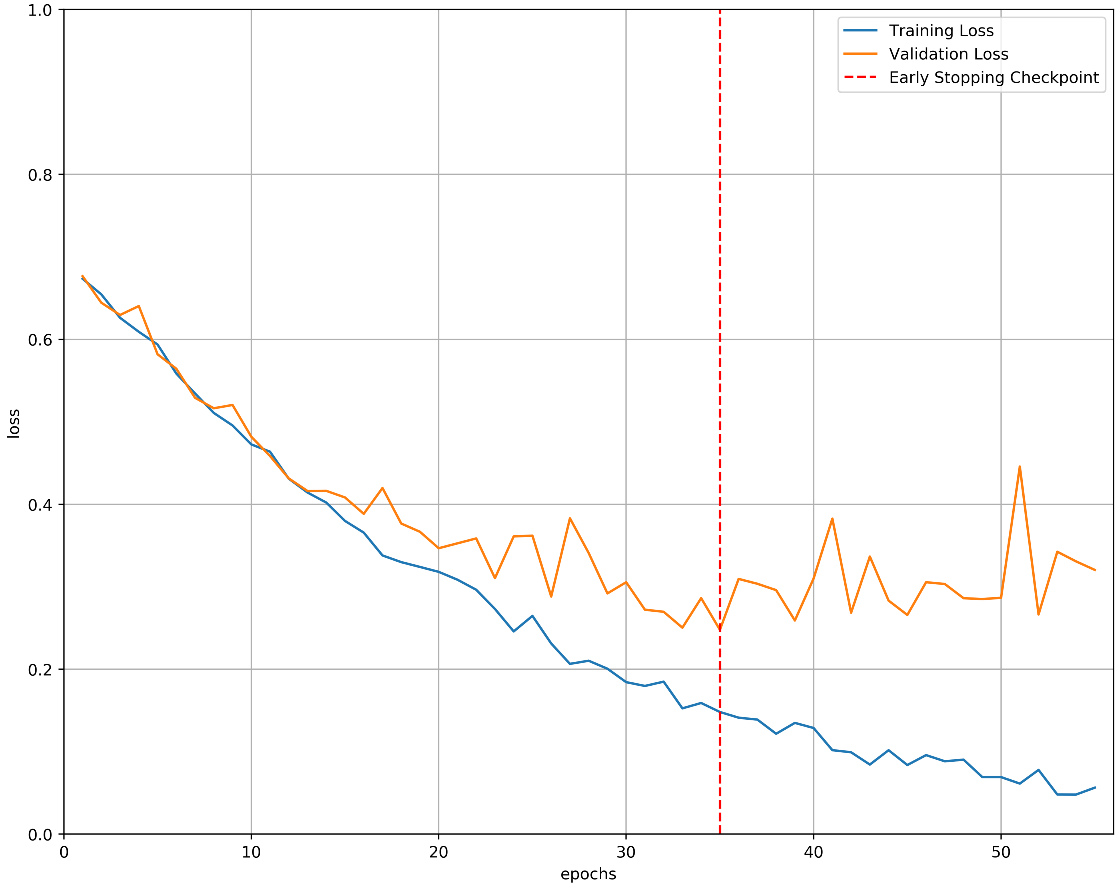

Supplementary Figure 12. Training and validation loss value changing along with the binary-class model training on Fold-2 subset. The position marked by the red dash line is the checkpoint of the final optimal model.

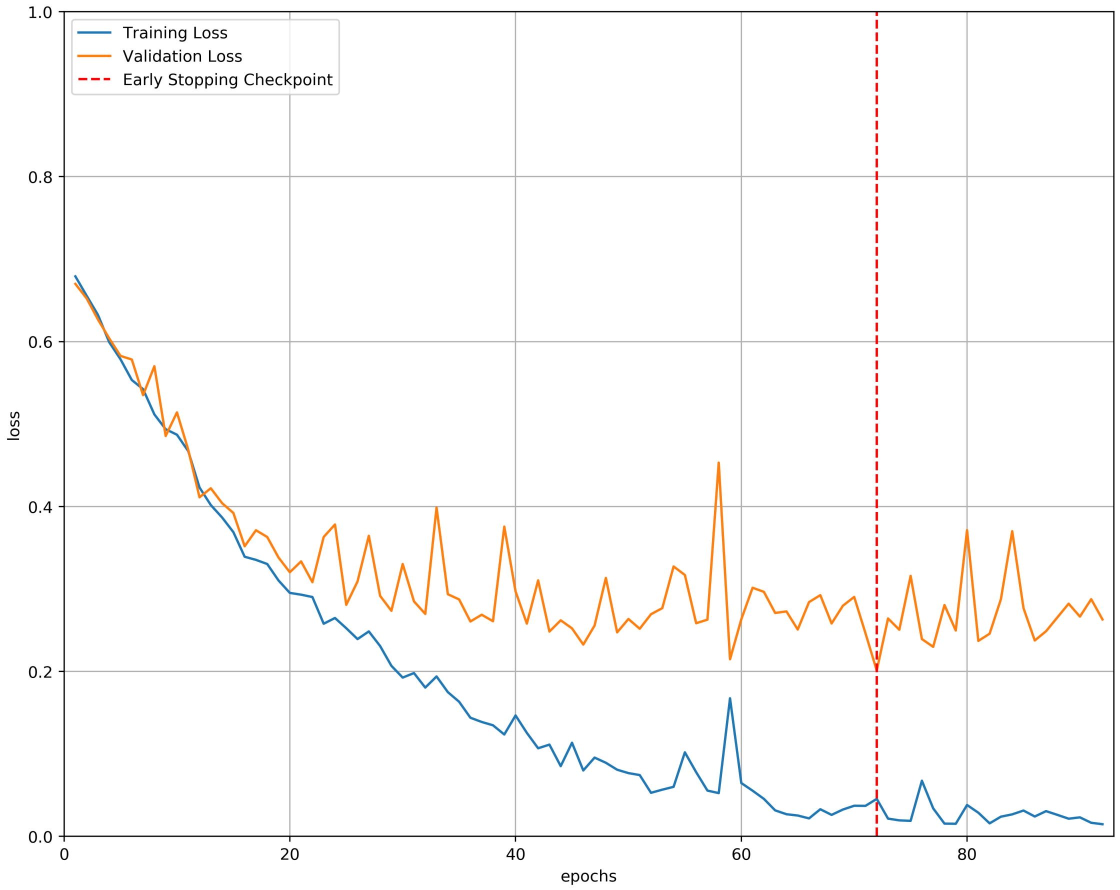

Supplementary Figure 13. Training and validation loss value changing along with the binary-class model training on Fold-3 subset. The position marked by the red dash line is the checkpoint of the final optimal model.

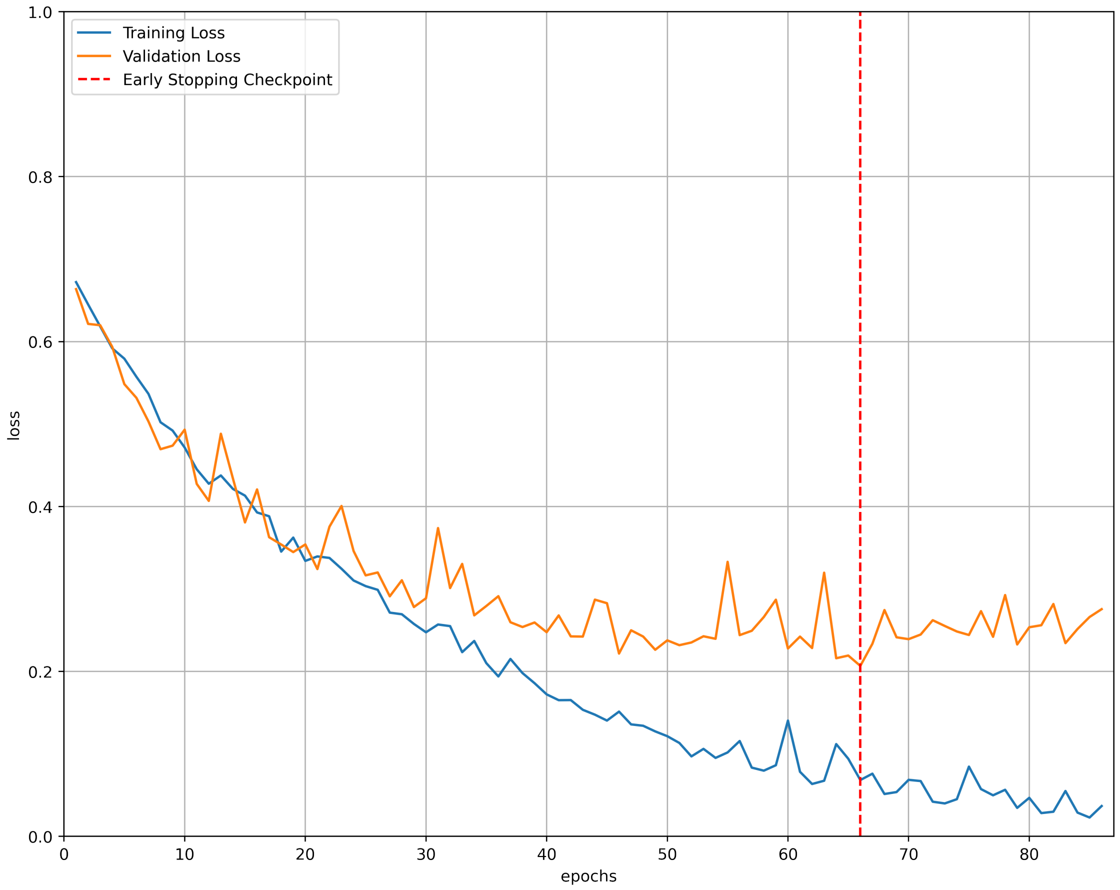

Supplementary Figure 14. Training and validation loss value changing along with the binary-class model training on Fold-4 subset. The position marked by the red dash line is the checkpoint of the final optimal model.

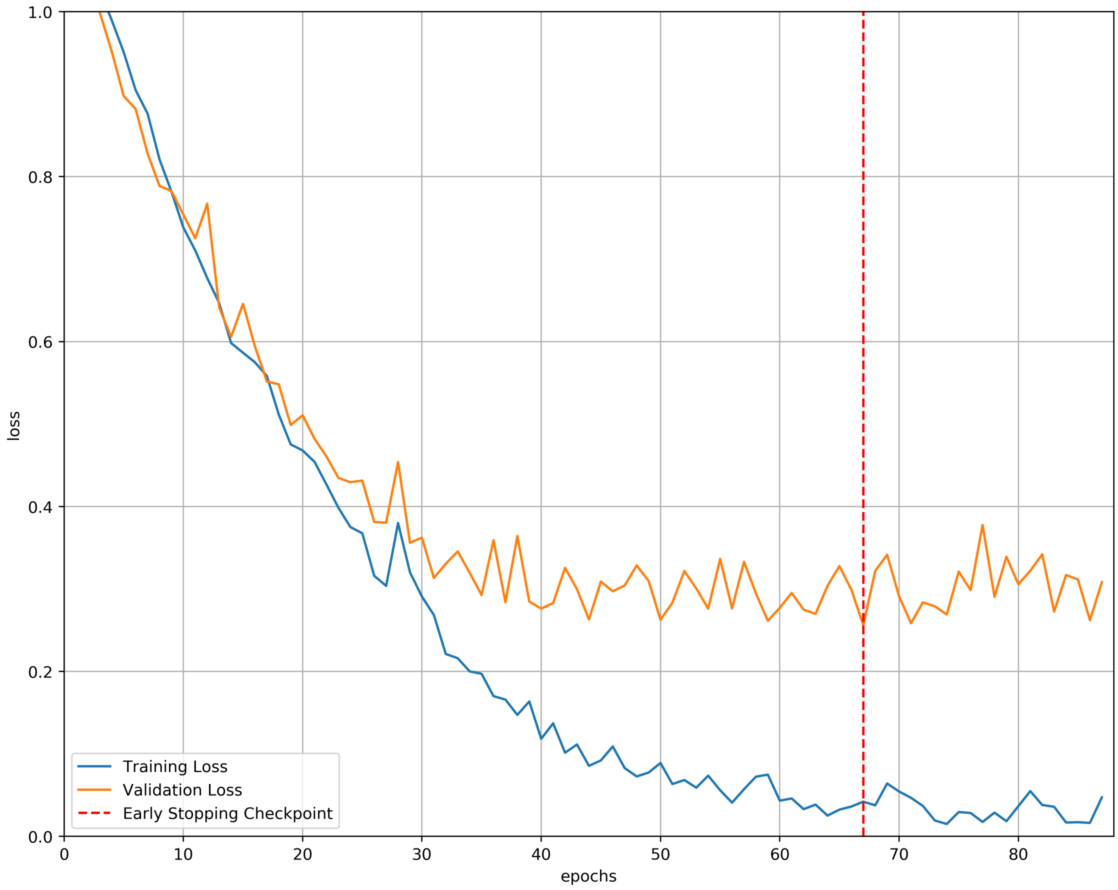

Supplementary Figure 15. Training and validation loss value changing along with the ternary-class model training on Fold-0 subset. The position marked by the red dash line is the checkpoint of the final optimal model.

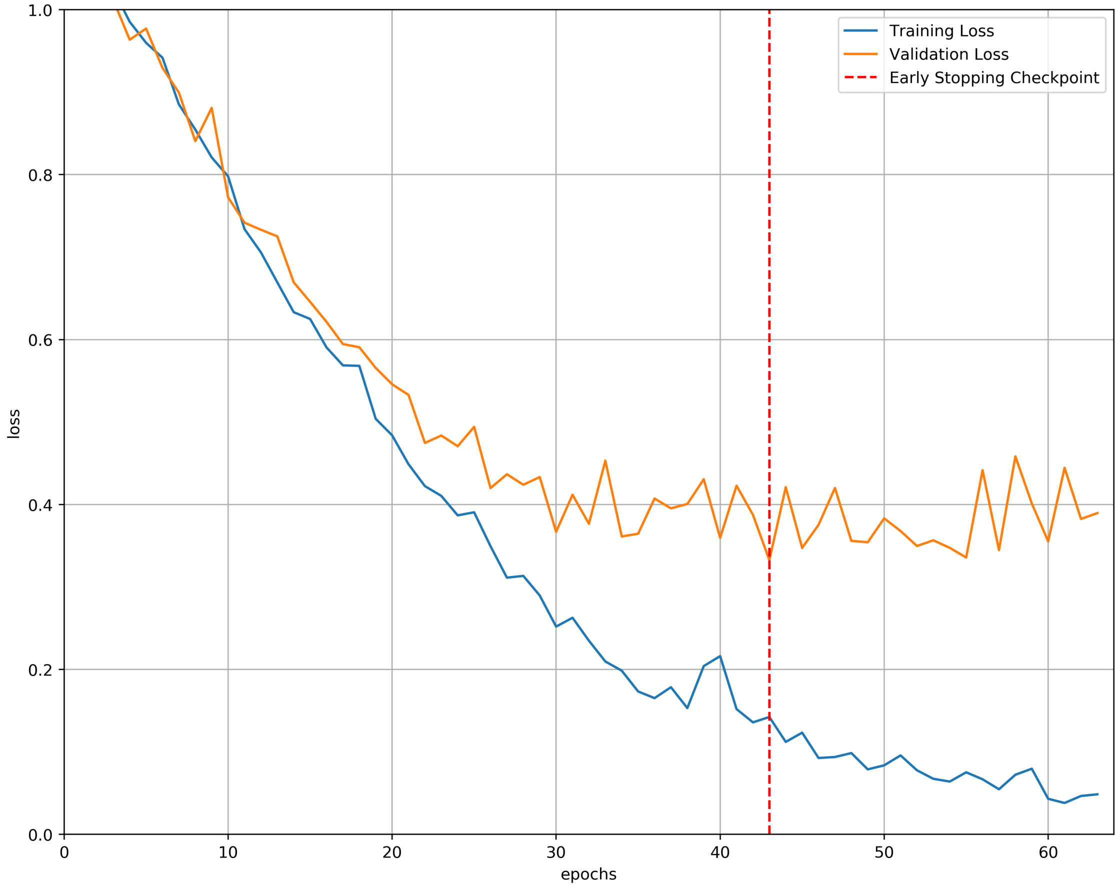

Supplementary Figure 16. Training and validation loss value changing along with the ternary-class model training on Fold-1 subset. The position marked by the red dash line is the checkpoint of the final optimal model.

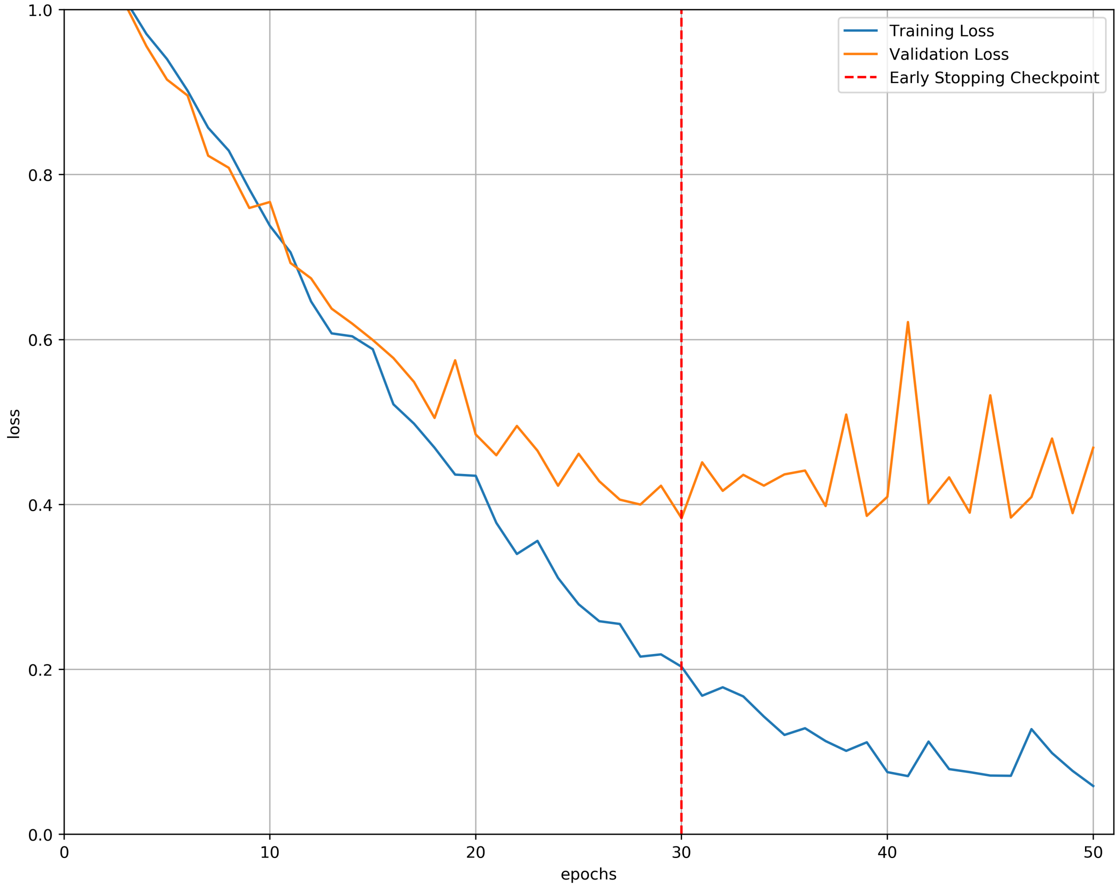

Supplementary Figure 17. Training and validation loss value changing along with the ternary-class model training on Fold-2 subset. The position marked by the red dash line is the checkpoint of the final optimal model.

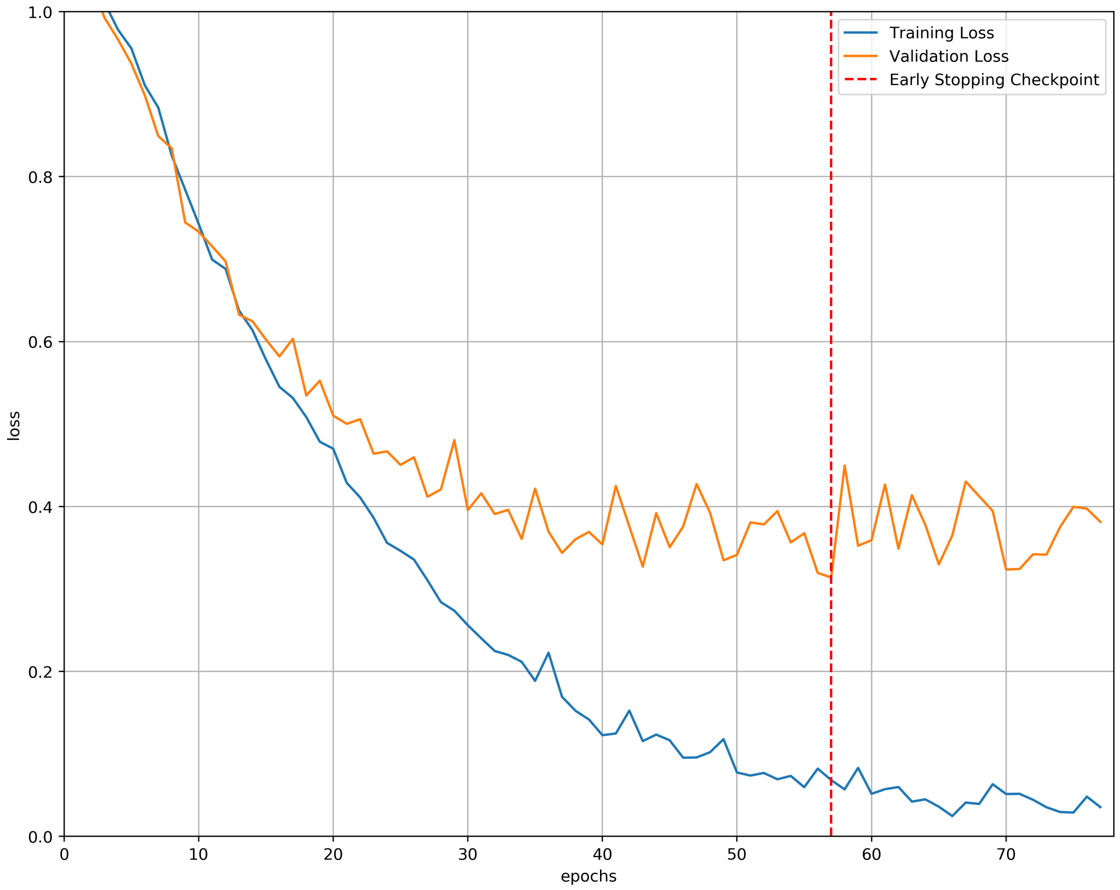

Supplementary Figure 18. Training and validation loss value changing along with the ternary-class model training on Fold-3 subset. The position marked by the red dash line is the checkpoint of the final optimal model.

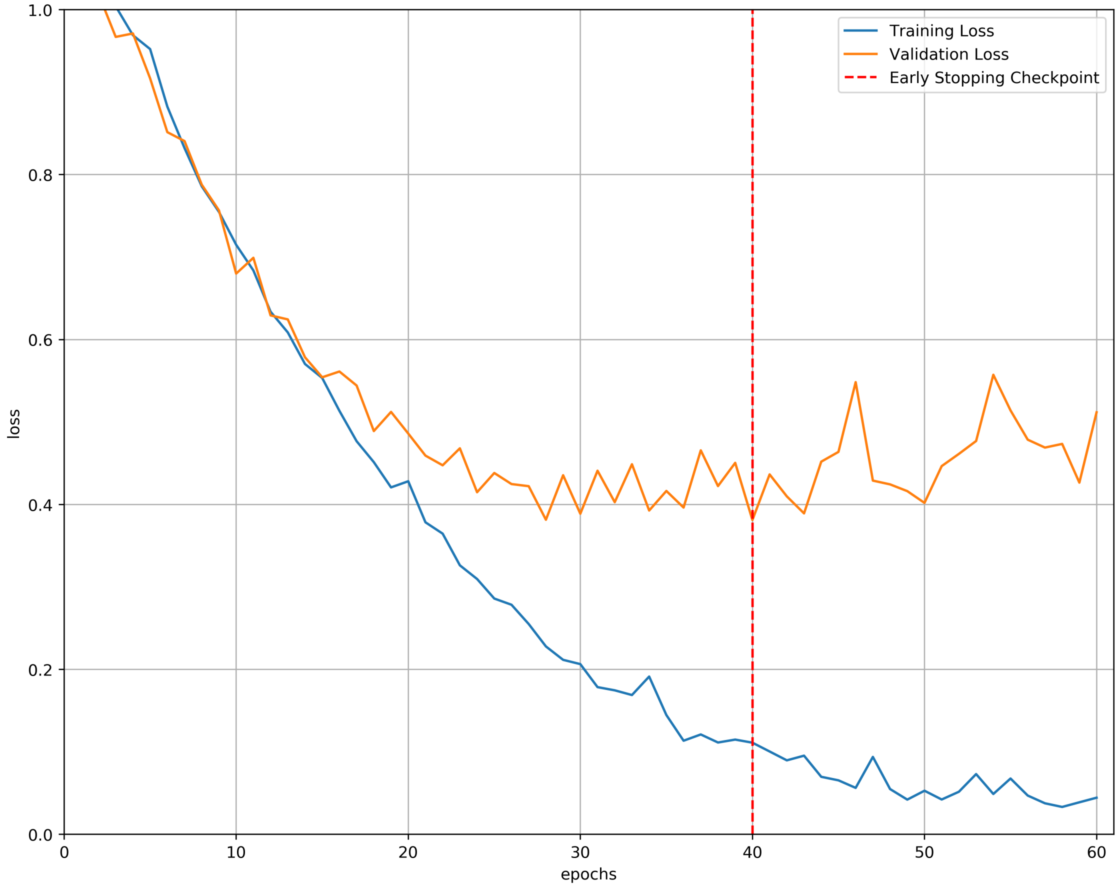

Supplementary Figure 19. Training and validation loss value changing along with the ternary-class model training on Fold-4 subset. The position marked by the red dash line is the checkpoint of the final optimal model.

References

1. Kipf, T. N. & Welling, M. Semi-supervised classification with graph convolutional networks. In *ICLR* (2017).

2. Xu, K., Hu, W., Leskovec, J. & Jegelka, S. How powerful are graph neural networks? In *ICLR* (2019).

3. Cangea, C., Veličković, P., Jovanović, N., Kipf, T. & Liò, P. Towards sparse hierarchical graph classifiers. In *Workshop on Relational Representation Learning (R2L) at NIPS* (2018).

4. Gao, H. & Ji, S. Graph U-Nets. In *ICML*, 2083–2092 (2019).

5. Knyazev, B., Taylor, G. W. & Amer, M. R. Understanding attention in graph neural networks. In *NeurIPS* (2019).

6. Lee, J., Lee, I. & Kang, J. Self-attention graph pooling. In *ICML*, 3734–3743 (2019).
