# Supplementary material for: Cell graph neural networks enable the precise prediction of patient survival in gastric cancer
Source: NPJ Precis Oncol. 2022 Jun 23;6:45. doi: 10.1038/s41698-022-00285-5 (PMC9226174; doi:10.1038/s41698-022-00285-5)
Supplement: Supplementary file 2 — REPORTING SUMMARY [file 41698_2022_285_MOESM2_ESM.pdf]

nature portfolio

Corresponding author(s): Pietro Lio, Dakang Xu, Geoffrey I. Webb, and Jiangning Song

Last updated by author(s): Feb 17, 2020

Reporting Summary

Nature Portfolio wishes to improve the reproducibility of the work that we publish. This form provides structure for consistency and transparency in reporting. For further information on Nature Portfolio policies, see our Editorial Policies and the Editorial Policy Checklist.

Statistics

For all statistical analyses, confirm that the following items are present in the figure legend, table legend, main text, or Methods section.

n/a	Confirmed	
☐	☒	The exact sample size (n) for each experimental group/condition, given as a discrete number and unit of measurement
☒	☐	A statement on whether measurements were taken from distinct samples or whether the same sample was measured repeatedly
☒	☐	The statistical test(s) used AND whether they are one- or two-sided<br>*Only common tests should be described solely by name; describe more complex techniques in the Methods section.*
☒	☐	A description of all covariates tested
☒	☐	A description of any assumptions or corrections, such as tests of normality and adjustment for multiple comparisons
☐	☒	A full description of the statistical parameters including central tendency (e.g. means) or other basic estimates (e.g. regression coefficient) AND variation (e.g. standard deviation) or associated estimates of uncertainty (e.g. confidence intervals)
☒	☐	For null hypothesis testing, the test statistic (e.g. F, t, r) with confidence intervals, effect sizes, degrees of freedom and P value noted<br>*Give P values as exact values whenever suitable.*
☒	☐	For Bayesian analysis, information on the choice of priors and Markov chain Monte Carlo settings
☒	☐	For hierarchical and complex designs, identification of the appropriate level for tests and full reporting of outcomes
☒	☐	Estimates of effect sizes (e.g. Cohen's d, Pearson's r), indicating how they were calculated

Our web collection on statistics for biologists contains articles on many of the points above.

Software and code

Policy information about availability of computer code

Data collection	HALO
Data analysis	Anaconda, Python, R

For manuscripts utilizing custom algorithms or software that are central to the research but not yet described in published literature, software must be made available to editors and reviewers. We strongly encourage code deposition in a community repository (e.g. GitHub). See the Nature Portfolio guidelines for submitting code & software for further information.

Data

Policy information about availability of data

All manuscripts must include a data availability statement. This statement should provide the following information, where applicable:

- Accession codes, unique identifiers, or web links for publicly available datasets
- A description of any restrictions on data availability
- For clinical datasets or third party data, please ensure that the statement adheres to our policy

The data used for the main analyses presented in this study are available for non-commercial use and can be obtained upon reasonable request.

Field-specific reporting

Please select the one below that is the best fit for your research. If you are not sure, read the appropriate sections before making your selection.

☒ Life sciences ☐ Behavioural & social sciences ☐ Ecological, evolutionary & environmental sciences

For a reference copy of the document with all sections, see nature.com/documents/nr-reporting-summary-flat.pdf

Life sciences study design

All studies must disclose on these points even when the disclosure is negative.

Sample size	The sample size of the mIHC dataset is 172. And each mIHC staining image contains around 7,000 -13,000 cells. In particular, we conducted the sub-sampling to enlarge the sample size of Cell-Graphs for model training. By treating each cell as a node in the Cell-Graph, we limited the graph size to no more than 100 nodes. A non-overlapping sliding window was then applied to extract the local regions that contained approximately 100 cells from each of the mIHC images. Consequently, we obtained 16951 Cell-Graphs for GNN model training and testing. According to the loss/accuracy change curves during model training, the dataset of this sample size was used to construct a stable GNN model.
Data exclusions	At the data preparation phase, only the patients labeled as “lost to follow up” were excluded from the dataset.
Replication	We conducted 5-fold cross-validation to validate the model's reproductivity. According to the results, the experiments are reproducible.
Randomization	The training, validation and holdout testing datasets were randomly split.
Blinding	Not applicable.

Reporting for specific materials, systems and methods

We require information from authors about some types of materials, experimental systems and methods used in many studies. Here, indicate whether each material, system or method listed is relevant to your study. If you are not sure if a list item applies to your research, read the appropriate section before selecting a response.

Materials & experimental systems

n/a	Involved in the study	
☒	☐	Antibodies
☒	☐	Eukaryotic cell lines
☒	☐	Palaeontology and archaeology
☒	☐	Animals and other organisms
☒	☐	Human research participants
☒	☐	Clinical data
☒	☐	Dual use research of concern

Methods

n/a	Involved in the study	
☒	☐	ChIP-seq
☒	☐	Flow cytometry
☒	☐	MRI-based neuroimaging
